# Revolutionizing cancer immunotherapy: unleashing the potential of bispecific antibodies for targeted treatment

**DOI:** 10.3389/fimmu.2023.1291836

**Published:** 2023-12-01

**Authors:** Xiaohan Guo, Yi Wu, Ying Xue, Na Xie, Guobo Shen

**Affiliations:** ^1^ West China School of Basic Medical Sciences and Forensic Medicine, Sichuan University, and Collaborative Innovation Center for Biotherapy, Chengdu, China; ^2^ State Key Laboratory of Biotherapy and Cancer Center, West China Hospital, Sichuan University, and Collaborative Innovation Center for Biotherapy, Chengdu, China

**Keywords:** bispecific antibodies, immunotherapy, targeted therapy, tumor microenvironment, cancer

## Abstract

Recent progressions in immunotherapy have transformed cancer treatment, providing a promising strategy that activates the immune system of the patient to find and eliminate cancerous cells. Bispecific antibodies, which engage two separate antigens or one antigen with two distinct epitopes, are of tremendous concern in immunotherapy. The bi-targeting idea enabled by bispecific antibodies (BsAbs) is especially attractive from a medical standpoint since most diseases are complex, involving several receptors, ligands, and signaling pathways. Several research look into the processes in which BsAbs identify different cancer targets such angiogenesis, reproduction, metastasis, and immune regulation. By rerouting cells or altering other pathways, the bispecific proteins perform effector activities in addition to those of natural antibodies. This opens up a wide range of clinical applications and helps patients with resistant tumors respond better to medication. Yet, further study is necessary to identify the best conditions where to use these medications for treating tumor, their appropriate combination partners, and methods to reduce toxicity. In this review, we provide insights into the BsAb format classification based on their composition and symmetry, as well as the delivery mode, focus on the action mechanism of the molecule, and discuss the challenges and future perspectives in BsAb development.

## 1 Introduction

Currently emerging cancer immunotherapies include cancer vaccines, T cell receptor T cells (TCR-T) or chimeric antigen receptor T cells (CAR-T), cytokine therapies, immune checkpoint blockades (ICBs), and tumor-targeted antibodies (1). Monoclonal antibodies (mAbs), in particular, are powerful tumor-targeted antibodies that have been licensed for use in cancer in the US and Europe for the first time in 2022 (2). However, the complicated pathophysiology of tumors limits the therapeutic efficacy of mAbs (3, 4), while the combination of two or more mAbs may be subject to safety and efficacy issues (5). Bispecific antibodies (BsAbs) have been developed to bind two specific epitopes or target proteins at the same time. These antibodies have improved specificity, increased targeting ability, and reduced off-target toxicity. Moreover, BsAbs have the potential to effectively lower the cost of treatment, revitalizing the field of cancer immunotherapy (6).

**Figure 1:**
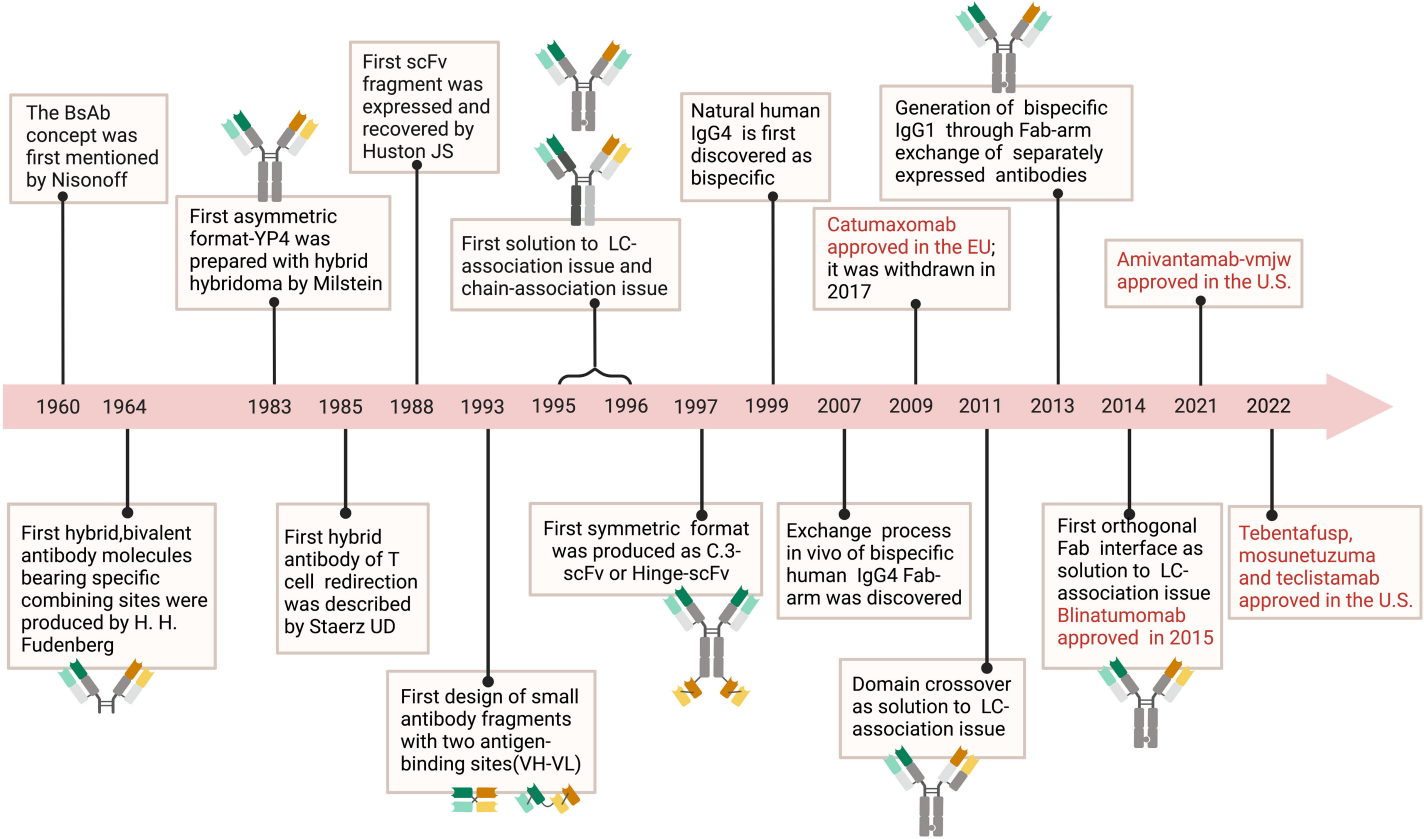
Timeline. The timeline showcases the technical innovations and clinical research in tumor of BsAb. In 1960, the concept of BsAb was proposed (11). In 1964, researchers created molecules with two different binding sites (12). BsAb with asymmetric structure was produced using hybridoma technology in 1983 (13). In 1985, the idea of BsAb that can redirect T cells was proposed (14). Diabody, a small molecule BsAb fragment, was designed in 1993 (15). In 1988, researchers developed scFv fragments (16). From 1995 to 1996, the problem of protein subunit pairing was first solved (17, 18). In 1997, BsAb with symmetric structure was manufactured (19). It was discovered in 1999 that natural human IgG4 molecules were bispecific (20). In 2007, the process of Fab fragment exchange in human IgG4 was explained (21). Catumaxomab was approved by the EU in 2009 but later withdrawn in 2007 (22). In 2011, the problem of light chain pairing was solved through domain swapping strategy (23). Bispecific IgG1 was produced using Fab fragment exchange in 2013 (11). In 2014, the problem of light chain pairing was solved through orthogonal Fab fragments (24). In 2015, Blinatumomab was approved (24). Amivantamab-vmjw was approved in the U.S. in 2021, and in 2022, Tebentafusp, Mosunetuzuma, and Teclistamab were also approved in the U.S (25–27).

The first BsAb was created in the early 1960s and was based on mild reoxidation of binding fragments from two different polyclonal sera (7). Later, based on enzymatic digestion of hybridoma peptides, hybridoma technology allowed the chemical coupling of mAbs or fragment antigen-binding (Fab) fragments (8). With the rapid development of genetic engineering technology, the multifunctional BsAb formats received great attention in clinical application. Mechanically, BsAbs inhibit tumor progression directly or indirectly mainly by redirecting immune effector cells into tumors, delivering radioactive or drug payloads to cancer cells, targeting multiple signaling pathways, and so on. For example, BsAb drug catumaxomab, which contains anti-epithelial cell adhesion molecule (EpCAM) and anti-cluster of differentiation 3 (CD3) molecule, destroys tumors via T cell-driven lysis, cytotoxicity triggered by antibodies, and phagocytosis via helper cells with Fcγ receptors (FcγRs) (9, 10). Four BsAb medications, including tebentafusp, faricimab, mosunetuzumab, and teclistamab, were approved for marketing in 2022 alone, suggesting that BsAbs are promising approaches to develop antitumor therapies (**Figure 1**).

In the review, we will systematically cover the antitumor principle and clinical applications of BsAbs in multiple formats. We will also introduce the preparation technology and delivery method of BsAb, and discuss their challenges and prospects in the treatment of solid tumors.

## 2 Format of BsAbs

The power of BsAbs lies in their capacity to create new activities that demand the union of two binding specificities in a single molecule (28). Their functionality can be greatly impacted by domain composition or “shifting” linker length and unique arrangements of (non-)chemical bonds. The design of BsAbs’ forms can also affect other factors including diffusion and pharmacokinetic activity (29, 30). In addition to expression platform’s stability and output, the presence or absence of undesirable side products is another factor that must be taken into account. The wide range of BsAb formats produced by the numerous designing methods can be categorized by their design elements or functional characteristics (28).

### 2.1 Classification of BsAbs based on composition

Based on structural components, BsAbs could be roughly classified into BsAbs with Fragment crystallizable (Fc) regions and BsAbs without Fc regions. BsAbs with Fc regions can help activate the immune system via Fc domain’s interaction with cell surface receptors, as well as endow the BsAb molecules with longer half-lives on account of their larger sizes and the neonatal Fc receptors (FcRn)-mediated recycling pathway (31). However, Fc region’s engagement with FcγRs can lead to serious cytotoxicity events, which may be a merit of BsAbs without Fc region (32). The Fragment variable (Fv)-only molecules are also easier to produce. While BsAbs without Fc domains lack interactions with CH (constant heavy chain)1/CL (constant light chain) regions, more techniques must be applied to stabilize the Fab regions.

#### 2.1.1 BsAbs with Fc regions

BsAbs that contain the Fc region include Immunoglobulin G(IgG) constructs such as Duobody (controlled Fab-arm exchange technology) (33, 34), Fabs-In-Tandem Immunoglobulin (FIT-Ig) (35), Cross-Mab (36), scFv-Fc constructs (single chain variable fragment), VHH-Fc constructs, and dual-affinity retargeting (DART)-Fc constructs (37).

Fc region offers BsAbs a number of advantages. The engagement of Fc region with membrane Fc receptors (FcRs) and certain complement system proteins help to activate the immune response. The Fc-directed receptor downregulation and malignant cells apoptosis through monocyte/macrophage trogocytosis is required for the antitumor efficacy of amivantamab (38, 39). These BsAbs have longer half-lives because of their big size and recycling pathways controlled by FcRn (37). An entire IgG antibody has a molecular mass of 150 kDa and is removed by the liver, whereas molecules with a molecular weight less than 60 kDa are filtered by the renal system (40). The combination of homologous variable heavy chain (VH) and variable light chain (VL) domains is further driven by the fusion of a heterodimerizing Fc region, making purification with affinity resins like protein G feasible (41).

However, off-target cytotoxicity and reduced treatment efficiency are associated with Fc-mediated downstream actions. When Fc region of medicinal antibodies interact with FcγRs, serious adverse effects may occur (42). Except for safety concerns, CD3-directing BsAbs with an active core demonstrated less effective *in vivo (* 28, 43). To reduce the aforementioned negative effects, presently available BsAbs targeting CD3 either omit the Fc region or have modified Fc domains to minimize FcγR interaction (28).

#### 2.1.2 BsAbs without Fc regions

BsAbs without Fc region lack the Fc-mediated effector actions mentioned above, but they aid in eliminating the chain-association problem. Moreover, the formats can be produced economically and high-yieldingly by expressing 1–2 peptides strands in simple eukaryotic and prokaryotic protein synthesis platforms (28, 44, 45).

BsAbs without Fc region mainly consist of tandem single-chain variable fragments (scFv2, taFv), bispecific one-domain antibody hybrid proteins, diabodies, and fragment antigen-binding (Fab fusion protein) (30). The taFv, which stands for the minimum BsAb, can be created by joining two scFvs together with a linker and normally ranges 50-60 kDa in size (30). However, these Fv-only moieties are short of the native-like connections with CH1/CL regions which is required for the stability and solubility of Fab regions (46). By creating a disulfide connection between the VH and VL domains, the stability of tandem scFv can be enhanced (30, 47). Bispecific single-domain antibody hybrid molecules can be made by one-domain antibodies, such as VH or VL domains, VHH, variable new antigen receptor (VNAR) and nanobodies (Nbs) (30). Compared to human programmed cell death-ligand 1 (PD-L1)-targeting mAb or vascular endothelial growth factor receptor type 1 domain 2 (VEGFR1D2) fusion protein alone, the HB0025 that combines the VEGFR1D2 and anti-PD-L1 mAb was more effective at preventing the growth of tumor (48). Diabody is a noncovalent heterodimer comprising the VH and VL portions of the scFv fragment linked by a short peptide. Since only some of the potential arrangements and orientations preserved binding potential for both antigens, it is crucial to choose the ideal VH/VL organization and alignment (30, 49, 50). In addition to domain order, the diabody-Ig platform utilize the dimerization domains CH1/CL, heterodimerizing EH Domain Containing 2 (hetEHD2), EH Domain Containing 2 (EHD2), and IgM heavy chain domain 2 (MHD2) to stabilize the diabody (51, 52). Furthermore, the domain connection was modified to promote heterodimerization (30). Fabs can serve as the foundation to which other binding elements are attached (30). A scFv may be attached to the C-terminal of either the light strand or the VH-CH1 (Fd) chains (e.g., bibody Fab-L-scFv, Fab-H-scFv), or to both strands (e.g., tribody, Fab(scFv)2) when Fabs are connected by hinge-regions (30, 45, 53–59).

The antigen-combining abilities of heavy-chain antibodies are entirely preserved in Nbs created from variable heavy-chain segments (VHH) in camelid heavy-chain antibodies (60). The molecule weight of the Nbs is 12–15 kDa, which is considerably less than the molecule weight of typical antibodies (150 kDa) (61, 62). Nbs with hydrophilic interfaces prevent the discrepancies in the heavy and light chain pairing of traditional antibodies, are not bound to light chains, which are vulnerable to polymerization, and are distinguished by tiny molecular mass, excellent solubility, and persistence (62). Nbs exhibit lower immunogenicity and simpler for humanizing and application in the clinic than traditional antibodies (62, 63). BsAbs can be created by modifying two Nbs which hit separate tumour antigens in order to enhance the selectivity of anticancer antibodies and render them optimal antibodies. In the detection and management of infection, cancer, and immunity, BsNbs are a scientific focus due to the improvement of BsNbs binding capacity, lengthening of plasma half-life, decrease in drug resistance, and severe side effects (62, 64). Liu et al. created the anti-CD20/CD3 BsNb by merging the anti-CD20 VHH gene with a thoroughly validated anti-human CD3 VHH built on the acquired anti-CD20 Nbs. After being incubated with human sera at 37°C for 48 hours, the anti-CD20/CD3 BsNb was still able to retain 80% of its binding efficacy. The findings demonstrated the potent anticancer activity of the developed anti-CD20/CD3 BsNb (62). Employing a BsNb which could concurrently target epidermal growth factor receptor (EGFR) and human epidermal growth factor receptor 2 (HER2) on cancer cells, Hong et al. created a dual-directed non-IgG form of BsAbs. The absence of Fc effector functional capabilities was restored by site-selective alteration of the EGFR-HER2-targeted BsNb employing the rhamnose (Rha) hapten through sortase A-regulated binding. The adjusted BsNb-Rha combination demonstrated significantly better pharmacokinetics and effective inhibiting actions against *in vivo* development of xenograft tumors (65). With the application of in silico methods, an improved bispecific design was created that can engage the therapeutically important antigens TNF-α and TNF-α-23 concurrently and, thanks to its increased avidity, efficiently block the death of TNF-α-sensitive L929 cells (66).

### 2.2 Classification of BsAbs based on symmetry

**Figure 2:**
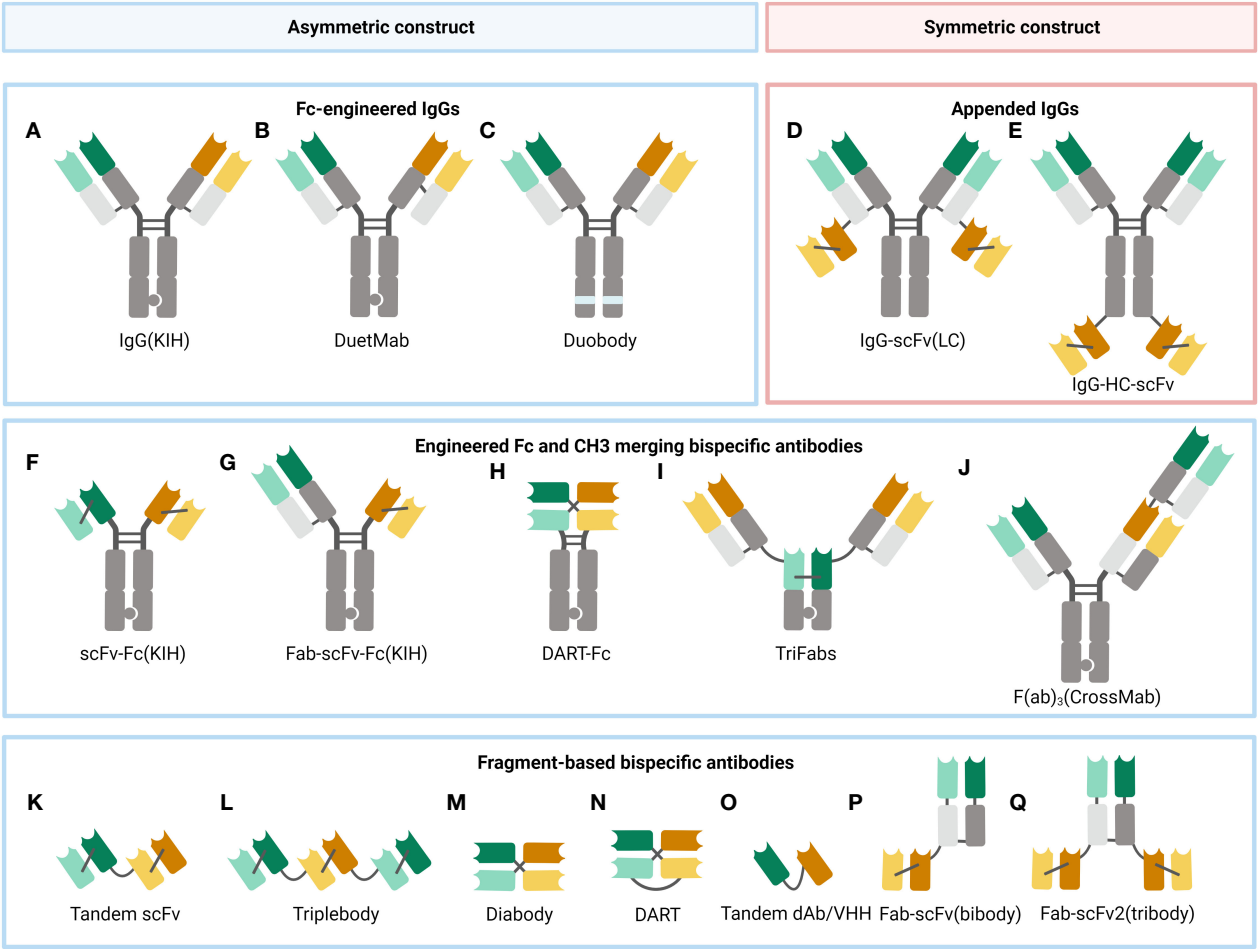
A selection of some common BsAb formats. **(A)** Fc-modified IgG format, built with the KIH technology to heterodimerize two different heavy chains. **(B)** DuetMab, improving the efficacy of homologous heavy and light chain coupling by designing a new disulfide bond to substitute the natural one in one of the CH1-CL interfaces (67). **(C)** Duobody, its Fc region was suppressed by inserting mutations, which circumvents the Fc-mediated cytotoxicity (68). **(D)** Appended IgG format, IgG- single-chain variable fragment (scFv) (light chain, LC). **(E)** Appended IgG format, IgG-heavy chain (HC)-scFv. **(F)** scFv-Fc format, constructed with the KIH approach. **(G)** Fab-scFv-Fc format, built with the KIH method. **(H)** DART-Fc construct, a DART protein unites two separate antigen-binding regions in a stable, diabody-like architecture (69). **(I)** TriFabs, IgG-based BsAbs made up of two normal Fab arms connected by flexible linker peptides to a third Fab-sized interaction unit (70). **(J)** CrossMab, antibody domain crossover enables the proper connection of generic light chains (71). **(K)** Tandem scFv (taFv), the minimum BsAb. **(L)** Triplebody, a construct similar to taFv. **(M)** Diabody (db), a short protein linker joins the heavy chain variable (VH) and light chain variable (VL) domains of a scFv segment to form a noncovalent heterodimer. **(N)** DART, made up of two Fv segments that heterodimerize to generate two distinct antigen-binding sites. **(O)** Tandem single-domain antibody (dAb)/VHH, made up of the antigen-engaging portion of heavy chain-only antibodies (72). **(P)** Fab-scFv (bibody), a scFv segment is fused to the C-terminus of the Fab framework to produce the bibody. **(Q)** Fab-scFv (tribody), a format similar to the bibody.

**Figure 3:**
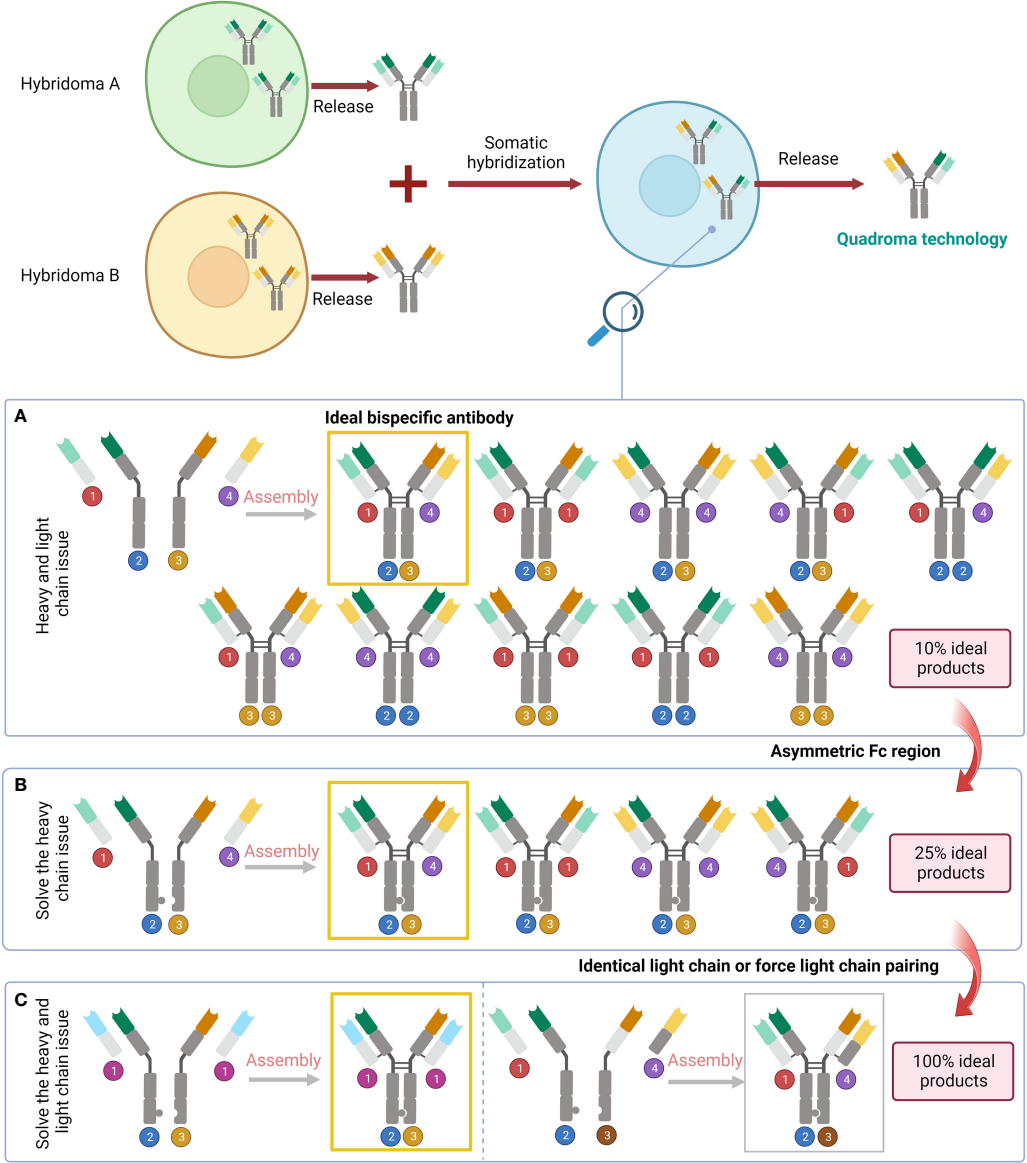
Problems raised by the variety of pairings and the corresponding solutions. **(A)** The result of simply fusing two different cell lines. **(B)** Solve the heavy chain problem by designing an asymmetric Fc region. **(C)** Address the issue with light chains by subsequent design of the identical light chain or force light chain pairing.

Through the lens of symmetry, BsAbs can be categorized into the asymmetric ones and the symmetric ones (**Figure 2**). Asymmetric BsAbs are initially created by combining two antibody-producing cell lines; with the advancement of genetic engineering, the technology is employed to produce BsAb, greatly assisting with the “chain” problems (it will be clarified in the following text) (**Figure 3**). Another approach to get around the “chain” issues and make the construction simpler is to design symmetric BsAbs, which could be generated by fusing or modifying IgG proteins.

#### 2.2.1 BsAbs of an asymmetric architecture

Every bispecific IgG molecule (antibody that is similar to natural immunoglobulins in constitute) is bivalent and has an asymmetric architecture because it contains at least distinct Fv regions (30).

Asymmetric BsAbs can be generated by merging two antibody-generating cell lines, e.g., producing a hybrid-hybridoma by fusing YTH12 and the MGICD19 cell lines (73). However, nine unwanted products will be generated by simply fusing two cell lines together (30). Genetic engineering is an alternative to remedy the issue. Through genetic methods, it is possible to create cell lines that produce two separate heavy and light chains and enable their proper integration (30). The heavy chain issue can be handled by BsAbs featuring an asymmetric Fc domain. Several methods created over the past 20 years leverage specific interchain disulfides, as well as steric or electrostatic steering effects to create a complimentary interface, benefiting heterodimerization against homodimerization (30). The knobs-into-holes (KIHs) strategy is a promising way to create BsAbs by inducing heterodimerization with mutations in the CH3 domain of each half antibody (74, 75). It was found that there was no significant change in the conception kinetics of BsAb produced by the KIHs technique, and the stability was similar to that of the wild-type antibody structure (74). Epcoritamab which recognises CD3 and CD20 and was created via cFAE of a humanized CD3 mAb and the human CD20 mAb7D8 (68). In extremely resistant patients with large B-cell lymphoma, notably those who had previously been exposed to CAR T cells, subcutaneous Epcoritamab produced profound reactions as well as reasonable safety (76). Flexible linker peptides may also be employed to fuse Fabs at their C-termini to a highly hereodimerizing Fab-like molecule (30). For instance, TriFabs are BsAbs with an IgG structure made of two conventional Fab connected by elastic linker protein to a single asymmetrical third Fab-sized interaction unit. The third module is S354C-Y349C disulfides connect CH3 knob-hole heterodimers, which replaced the original Fc region (70).

To maintain the related functions and favorable qualities, the major methods to create the forms in the category aim to preserve the structure of natural antibodies precisely. Nevertheless, in certain formats, the complex architecture to address the chain-association problem may negate these benefits (28). Compared to formats that permit multivalent target binding, asymmetric forms’ lower avidity may influence their strength (28, 77).

#### 2.2.2 BsAbs of a symmetric architecture

Incorporating two particularities into single heavy & light combination or peptide strand will result in symmetrical BsAbs, and can solve the chain-association problem whilst preserving the Fc domain (28). Also, the symmetric form is easier to construct (58).

One strategy is to produce IgG fusion proteins, to be more specific, by fusing scFv, domain antibodies and scaffold proteins, Fab arms, or additional VH and VL domains. For instance, the T cell-stimulating BsAb CLN-049, which binds to CD3 and FLT3, was created as an IgG heavy chain/scFv hybrid (78). Another strategy is to modify IgG molecules. Either the VH and VL domains’ original antigen-binding sites was altered or an extra binding site was transplanted to the Fc fragment’s bottom portion (30). With two unique, regionally separated interaction sites inside the human antibody CDR loops, dual targeting Fab (DutaFab) molecules was developed (79).

While almost mimicking natural antibodies, symmetric forms bear differences in size and organization. These variations may adversely alternate antibodies’ features (eg, consistency and solubility), which could disrupt their physicochemical and/or pharmacokinetic qualities (28, 80, 81). Most clinical test formats feature tetravalent 2+2 configurations due to the symmetric character and thus anticipated to have enhanced affinity against both malignant cells and T cells. However, this was just of secondary significance in terms of therapeutic efficacy. Despite sharing identical neoplasm binding and having improved T cell interaction compared to 2+2, 2+2B (Bispecific T cell engager (BiTE)-Fc) and 2+2HC (IgG-[H]-scFv) both failed to exhibit anticancer efficacy *in vivo *(28, 58).

## 3 Preparation technology of BsAbs

Methods of preparation of BsAbs are classified as chemical coupling, hybridoma binding and gene cloning methods (82, 83). Chemical cross-linking is the process of forming disulfide bonds between antibody molecules of different specificities or F(ab’) by using a specific chemical cross-linking agent, thus creating a heterodimer. This can be in the form of coupling between whole antibody molecules, or between F(ab’) and F(ab’)2 (84). BsAbs prepared by this method can directly utilise existing antibodies and has a high yield, but its activity may be affected by damage to the antigen-binding site (85). Hybridoma technology is based on monoclonal antibody technology, in which hybridoma cells secreting two antibodies are fused to produce hybridomas that stably secrete BsAb. Co-expression of the respective immunoglobulin (Ig) genes produces two types of H and L chains, which combine to form a BsAbs with the characteristics of the parental Ig (86). BsAb prepared using the hybridoma method is more random and relatively inefficient, but it has better biological activity and a more stable antibody structure (87). Genetic engineering techniques have opened up new avenues for the preparation of BsAbs, either by cloning the gene encoding the parental antibody and transfecting it into host cells for direct expression of BsAbs, or by gene shearing and constructing a scFv for the preparation of modified BsAbs (30).

A quality technology platform is key to the success of BsAbs development, and several technology platforms are in progress (88). Among the BsAbs technology platforms with Fc are Crossmab/KIH, DVD-Ig (dual variable domain-Ig), IgG-scFv, FIT-Ig, mAb-Trap, duobody ect (48, 89–92). Dual antibody technology platforms without Fc include BiTE, DART, TandAb, ImmTAC, BriKE, etc (93, 94). Developing BsAbs with the aforementioned functions requires careful adjustment of a number of variables in order to attain the ideal practical result. A blend of complementary binders and other elements in formats which allow the required functionality is necessary for the creation of BsAbs (95). Here, we will introduce several promising preparation methods.

### 3.1 Knob-into-hole technology

BsAbs possess two distinct paratopes on their variable regions that recognize two separate antigens, in contrast to normal mAbs that contain two same antigen-binding or Fab components. Due to this special characteristic, BsAbs can take on more intricate forms, such as homodimers made of two distinct arms or light chain mismatches, among others. The KIH configuration was employed to create BsAbs to foster heavy-chain heterodimer pairing of the two hemi-antibodies (17, 96). Knob and hole mutations shouldn’t affect antigen recognition or Fc activity since they are in the CH3 domain interaction surface. There are multiple ways to prevent light chain mismatching over assembly (97). One method involves expressing two half-antibodies in two separate host cells during an *in vitro* production process. Following two distinct Protein A specificity grab procedures, the two hemi-antibodies are combined for *in vitro* synthesis by reduction and oxidation, which is proceeded by subsequent BsAb purifying (98, 99). It was possible to detect chemical alteration sites and evaluate the steadiness and wholeness along with the operation of a BsAb by applying a variety of stressful situations together with dimension isolation chromatography, ion switch chromatography, LC-MS/MS peptides mapping, and practical examination by cell-based tests. Grunert et al. observed that IgG1 KIH CrossMab-engineered BsAbs were significantly more stable than commercially available antibodies (100). Furthermore, Liu et al. discovered that the KIH architecture did not significantly modify the organization or conformation motion, and the structural security is comparable to that of wild-type (WT) IgG4 (apart from a minor change in the CH3 domain) generated in *E. coli* (74).

The KIH structure and *in vitro* construction may effectively promote the heterodimerization of the heavy chains; nevertheless, throughout hemi-antibody isolation and BsAb installation, some homodimers (such as knob-knob and hole-hole dimerization) remain detectable (96). In the Fc region of an IgG1 BsAb, Elliott et al. uncovered the molecular specifics for KIH and homodimer engagements (101). The knob-knob and hole-hole Fc component homodimers’ X-ray crystal structures have been resolved, revealing a juxtaposed Fc configuration. Bispecific variations have been identified and quantified via intact mass evaluation (99, 102–104).

### 3.2 CrossMab technology

Combined with techniques that allow for accurate heavy-chain connection with already-existing pairs of antibodies, CrossMab technology, in conjunction with KIH technology, permits an appropriate antibody light-chain interaction with its corresponding heavy chain in BsAbs (105). The BsAbs are made up of two arms: one altered, and the other is not. Adjustments may be restricted to the VL-VH domain, the whole Fab region, or the CL-CH1 area (23). Due to the modifications, the intended chain interaction is enacted because the unaltered heavy chain could no longer interact with the altered light chain. In terms of structure and purity, the CL-CH1 CrossMAb displayed the best results among the three potential changes (106). Clinical trials are now being conducted to assess a number of BsAbs developed by CrossMAb innovation (71). The BsAbs that have currently been produced using CrossMAb include RO6958688 (CD3, CEA) for carcinoembryonic antigen (CEA)^+^ solid tumors, RG7221 (Ang2, VEGF), RO7121661 (PD-1, TIM3), and RO5520985 (Ang2, VEGF) (28, 107).

### 3.3 FORCE technology

Being a high-throughput method, Format Chain Exchange (FORCE) provides effective combined production of BsAbs in various arrangements for screening in ultimate form. The technique is based on the formation of BsAb from monospecific educts carrying various binders in various forms. Input agents for the production of BsAbs are monospecific entities with matching CH3-interface-regulated and imitation chains with affinity tags, analogous to KIH hemi-antibodies. These comprise mutations which result in minor interaction repulsions without affecting the production or biological characteristics of educts. Instability at the CH3-educt interfaces resolves to complete compatibility upon mild reduction of pairings of educts, initiating unprompted chain interchange events. This results in the formation of sizable BsAb matrices including various binders in various forms. Processing automation is made possible by benign biological characteristics, excellent production outputs of educts, and ease of purification. The monospecific input components comprise designed Fc-mimic chains that induce heavy-chain interchange processes that lead to formation. Production automation is made possible by efficiency, sturdiness, and simplicity (including assembly and one-step output purifying), allowing for thorough screens of BsAb binder-format layout spaces (95).

### 3.4 SEEDBodies technology

By creating strand-exchange engineered domain (SEED) CH3 heterodimers, Davis et al. built a heterodimeric Fc system which facilitates the construction of bispecific and asymmetric hybrid proteins. Human SEED CH3 heterodimers, which are made up of alternate parts of human IgA and IgG CH3 patterns, are produced by the variants of human IgG and IgA CH3 regions. When produced in mammal cells, the resultant pair of SEED CH3 regions selectively interacts to generate heterodimers. SEEDbody (Sb) fused proteins are made up of [IgG1 hinge]-CH2-[SEED CH3], and one or more fused couples could be genetically related to them. Mammal cells producing SEEDbody (Sb) fusing proteins result with large outputs of Sb heterodimers which can be easily separated to get rid of the modest byproducts. To simplify examination of heterodimer production in the current study, fusion companions are usually introduced to the N- or the C-terminal of one Sb chain. The lengthy plasma half-life prolongation characteristic of analogous fusion involving Fc segments and IgG1 standards were visible in the Sb pharmacokinetics after being delivered intravenously to rodents (108).

### 3.5 Duobody® technology

DuoBody® innovation was created by Engelberts et al. to produce complete IgG1 BsAb employing cFAE. Here, under carefully monitored fabrication circumstances, two original IgG1 mAb with paired single spot mutations in the IgG Fc region rearrange into full-length bispecific IgG1s (33, 109). At both the laboratorial and industrial scales, it was demonstrated that the cFAE technique is a simple and reliable way to produce durable BsAb with a greater output contrasted with other BsAb technologies (110). Additionally, the approach offers the chance to create and screen sizable and different arrays of BsAb, allowing for the identification of BsAb with the best functionality (68). In patients with progressed solid cancers, DuoBody-CD40-4-1BB has the potential to improve anticancer immunity by altering DC and T-cell activities (111). A transformed CD3 mAb and the human CD20 mAb 7D8 were combined to create DuoBody-CD3xCD20 (GEN3013), a BsAb recognizing CD3 and CD20 (76, 112). The subcutaneous injection route might offer a way to lower patients’ peak cytokine levels, as well as a way to ease their medical strain and make more efficient use of the facility’s resources (68).

## 4 Delivery of BsAbs

**Figure 4:**
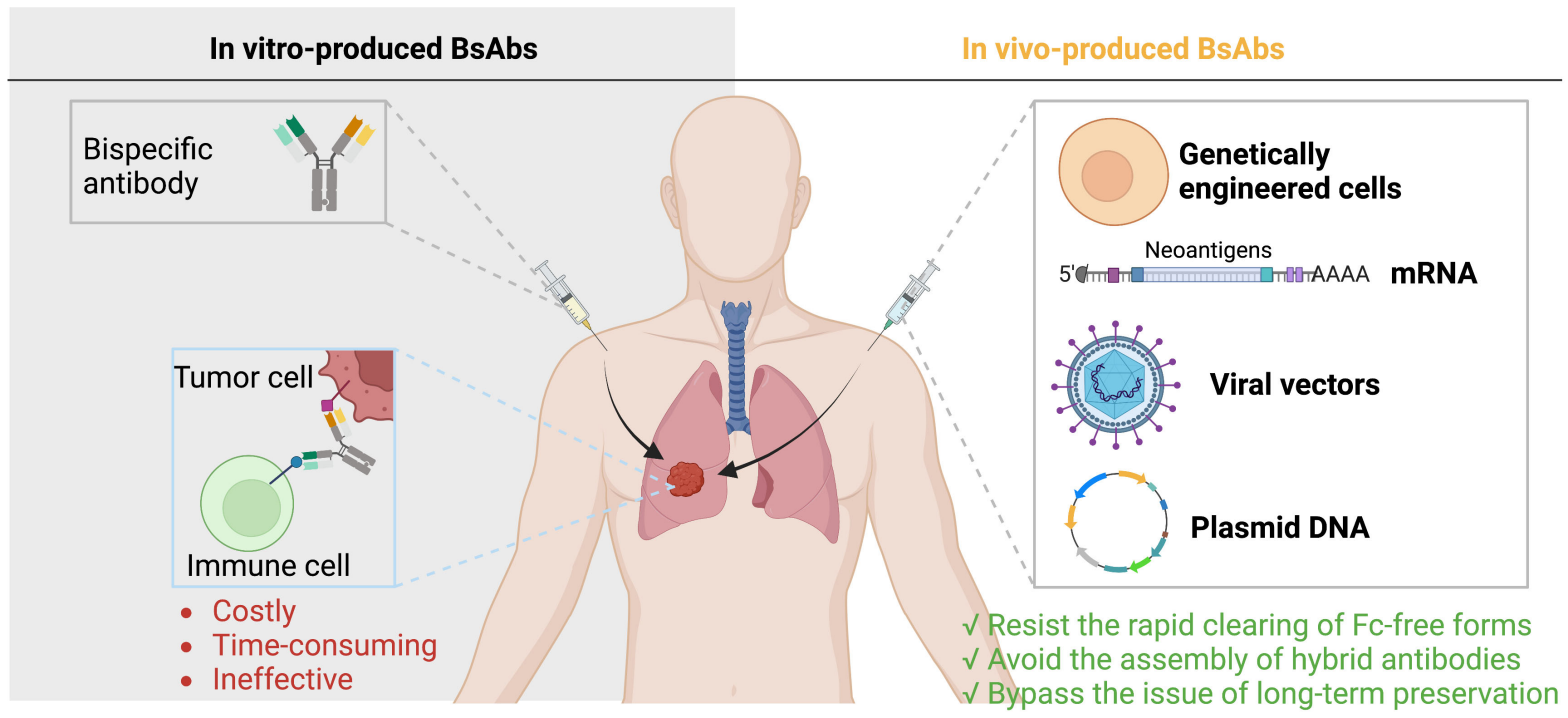
Delivery of BsAbs. There are two ways to deliver BsAbs to tumors: administer pre-made BsAbs, which is expensive and inefficient, or allow for *in vivo* synthesis of BsAbs, which can avoid elimination assembly, and preservation issues.

Currently, there are two ways to deliver BsAbs to the tumor sites. The first is to administer BsAbs after they have been produced *in vitro*, a process that is generally costly, time-consuming, and ineffective. The second is to enable the *in vivo* synthesis of BsAbs, which can counteract the quick kidney elimination of Fc-free forms, rendering a prolonged potent antibody level and can bypass issues with recombinant antibody assembly and long-term preservation (113, 114) (**Figure 4**).

### 4.1 Delivery of *in vitro*-produced BsAbs

The majority of antibody-based treatments are administered following *in vitro* synthesis. In terms of the scope of action, these deliveries could be classified into circulatory delivery (e.g., intravenous (IV), intracutaneous (IC), subcutaneous (SC), or intramuscular (IM) delivery) and local delivery. Delivery to cancer is regulated by an intricate interaction of factors: the spot of infusion (e.g., IV, IC, or intratumor), carry via the plasma and lymphatics, permeation across the endothelium and basal lamina into the interstitium, hydrodynamic tension in the blood *vs*. the carcinoma tissues, and removal of biologics from the scheme (e.g., by renal filtration, hepatic evacuation (115)).

Although the IV method provides 100% bioavailability, physiological obstacles and circulatory dispersion significantly lower the real BsAb level in the target sites (116). Additionally, IV infusion is inconvenient and takes time, as purified BsAbs that require higher concentrations must be supplied by gradual IV in order to prevent injection responses (114). Considering the maximal amount of infusion is limited, accordingly, low BsAb solubility at high densities is the most typical barrier for SC or IM. Clinical-grade BsAbs are very costly and have production difficulties, such as low durability over long-range preservation and a propensity to congregate (117, 118). The BBB is a tough hurdle that prevents drug transport to the brain. The employment of intrinsic brain endothelial delivery channels is a viable strategy to bypassing the biological hurdle via receptor-mediated transcytosis (RMT). BsAbs are the perfect choice for the purpose since treatments engineered demand at least two capabilities, one that aids delivery and the other to give clinical effect (119). The most typical RMT (TfR, InsR, and LRP1 receptor) have been effectively exploited to cross the BBB (120, 121). Since the BBB is disrupted in brain malignancies, it is necessary to particularly look into the production of novel candidate receptors facilitating RMT particularly for the blood–brain tumor barrier (122). Besides getting to the pathways, innovations to effectuate BsAb distribution into the brain should also be taken into account (119).

Regional administration can improve potency and lessen overall contact for various ailments. In certain malignant situations, intratumoral or intraperitoneal injection of BiTEs may confine effects to malignancies (123–125). An efficient and feasible approach is to use solid implants, granules, or injected storage made of biodegradable and biocompatible polymers to entangle BsAbs and unleash them as the polymer breaks down. PEG-PLA copolymers, a depot-injectable polymeric platform created *in situ*, were utilized to transport BiJ591 (a BsAb targeting CD3^+^ T cell and prostate-specific membrane antigen (PSMA) on prostate cancer cell) against prostate tumor. The transport method could regulate BiJ591’s discharge while preserving its durability and activity, and the treatment efficiency was higher than IV delivery (126, 127).

Transport techniques utilizing nanoparticles also showed impressive performance. Contrasted to XA-1 protein alone, lipid nanoparticle-encased mRNA-expressed XA-1 displayed greater potential anti-cancer effectiveness (128). Administration platforms like liposomes and cell-infiltrating peptides have also been demonstrated to be more efficient than the use of a solitary drug (127).

### 4.2 Delivery of *in vivo*-produced BsAbs

*In vivo* gene treatment was created, in an effort to strike a balance between potency and security. The two major schemes are the *in vitro* inoculation of genetically engineered cells and straight gene transport via vectors, mRNA, and plasmid DNA (127).

Both viral and nonviral vectors could be employed to convey the genetic material encoded for BsAb *in vivo*, while utilizing mRNA or plasmid DNA, direct *in vivo* administration of synthesized nucleic acid-coded BsAbs suggests new methods (114, 117, 129, 130). A CaPO-nanoneedle/minicircle DNA platform generated BsAb (EpCAM/CD3-targeted) resulting in a considerable slowdown of tumor development and a prolongation of animal life-span with minimal toxicity in an intraperitoneal xenograft model with human ovarian carcinoma cell line SKOV3 (131). A synthesized HER2 plasmid DNA-coded BiTE effectively recruited T cells to identify and ruin HER2^+^ melanoma cells, and it exhibited potent anti-cancer effects *in vivo (* 127, 132). Additionally, numerous oncolytic viruses were equipped with expression cassettes generating BiTEs, proving that when straight oncolysis and T cell-regulated destruction are combined, anticancer potency is increased in contrast with the original equivalent in syngenic and xenograft malignancy models (114, 127, 129, 132). The strategy may speed up the clinical progression of new BsAbs as it is quick to produce pharmaceutical-grade mRNA and DNA. Moreover, the temperature tolerance of DNA could make it simpler to carry and administer to larger populations due to its long-term preservation and temperature durability. Also, the *in vivo* synthesis could maintain an efficacious protein level, allaying worries about a quick kidney clearance (129, 130, 133, 134).

It is possible to *in vitro* transmit genetic information into cells obtained from patients, after which the BsAb-releasing cells are infused back to the patient. Compared to straight gene transfer methods, tumor infiltration and general on-target/off-cancer cytotoxicity problems may be addressed by the tumor anchoring of injected cells and ensuing intratumoral release (114, 135). New methods centered on modified cells secreting BiTEs (STAb cells) natively are now being developed (135). Research detailed the creation of anti-CEA and anti-CD3 dAb-releasing human T cells and revealed that the intratumoral delivery of lentivirally transfected STAb-T cells dramatically decreased *in vivo* cancer progression in human HCT-116 colon malignancy xenografts (135).

## 5 Anti-tumor mechanism of BsAbs

BsAbs could executive its antitumor effect in three major ways, including redirecting immune effector cells to tumors, delivering radioactive or drug payloads to carcinoma cells, targeting multiple signaling pathways to suppress tumor progression directly or indirectly. The classification of BsAb clinical applications based on target antigens is presented in **Table 1**.

**Table 1:** Clinical-stage BsAbs for cancer indications.

Target	Interventions	Format	Conditions	Phase	Sponsors	NCT Number	Ref
Targeting immune effector cells: T cell							
CD3×CD19	AMG562	Fab-scFv (bibody) *P	DLBCL; MCL; FL	Phase 1	Amgen	NCT03571828	
	Blinatumomab	Tandem scFv *K	B-ALL	Phase 2	Amgen Research Munich GmbH	NCT01209286	
	A-319	scFv-Fab *P	DLBCL	Phase 1	EVIVE Biotechnology	NCT04056975	
	TNB-486	IgG4 *A	B-Cell Lymphoma	Phase 1	TeneoTwo Inc.	NCT04594642	
	AFM-11	scFv×scFv (diabodies) *M	Leukemia, B-Cell	Phase 1	Affimed GmbH	NCT02848911	
CD3×CD20	Plamotamab (XmAb13676)	Fab-scFv-Fc (KIH) *G	B-cell NHL; CLL	Phase 1	Xencor, Inc.	NCT02924402	
	Odronextamab (REGN1979)	IgG4 *A	NHL; CLL	Phase 1	Non-Hodgkin Lymphoma Chronic Lymphocytic Leukemia	NCT02290951	
	Epcoritamab (GEN3013)	IgG1 *A	LBCL	Phase 1	Genmab AbbVie	NCT05733650	
	FBTA05	IgG-HC-scFv *E	Leukemia; SCT	Phase 1 Phase 2	Technical University of Munich	NCT01138579	
	Mosunetuzumab (BTCT4465A)	IgG1 *A	BCL	Phase1	Hoffmann-La Roche	NCT04313608	
	GB261	Fab-scFv-Fc (KIH) *G	B-Cell NHL; CLL	Phase 1 Phase 2	Genor Biopharma Co., Ltd.	NCT04923048	
	Obinutuzumab (RO7082859)	Fab3CrossMab *J	DLBCL	Phase 2	Hoffmann-La Roche	NCT00576758	(136)
CD3×CD22	JNJ-75348780	IgG4 *A	Lymphoma, Non-Hodgkin Leukemia, Lymphocytic, Chronic, B-Cell	Phase 1	Janssen Research & Development, LLC	NCT04540796	
CD3×CD28	rM28	Tandem scFv *K	Malignant Melanoma	Phase 1 Phase 2	University Hospital Tuebingen	NCT00204594	
CD3×CD33	AMV564	diabody *M	AML	Phase 1	Amphivena Therapeutics, Inc.	NCT03144245	
	AMG330	Tandem scFv *K	AML, Myelodysplastic Syndrome	Phase 1	Amgen	NCT02520427	
	AMG673	Triplebody *L	Recurrent Squamous Cell Lung Carcinoma	NA	SWOG Cancer Research Network	NCT02154490	
	JNJ-67571244	IgG4 *A	Leukemia, Myeloid, Acute, MDS	Phase 1	Janssen Research & Development, LLC	NCT03915379	(137)
	GEM333	Fab-scFv-scFv *P	AML	Phase 1	AvenCell Europe GmbH GCP-Service International Ltd.	NCT03516760	
CD3×CD38	ISB 1342	Fab-scFv-Fc (KIH) *G	Relapsed/Refractory MM	Phase 1	Ichnos Sciences SA Glenmark Pharmaceuticals S.A.	NCT03309111	
	Y150	Fab-scFv-IgG1 *A	Relapsed/Refractory MM	Phase 1	Wuhan YZY Biopharma Co., Ltd.	NCT05011097	
	AMG424 (Xmab13551)	Fab-scFv-Fc (KIH) *G	Relapsed/Refractory MM	Phase 1	Amgen	NCT03445663	
CD3×CD123	Vibecotamab (XmAb14045)	Fab-scFv-Fc (KIH) *G	AML MDS	Phase 2	M.D. Anderson Cancer Center	NCT05285813	
	JNJ-63709178	IgG4 *A	Leukemia, Myeloid, Acute	Phase 1	Janssen Research & Development, LLC	NCT02715011	
	Flotetuzumab (MGD006)	DART *N	AML	Phase 1 Phase 2	MacroGenics	NCT02152956	
	APVO436	SCFV-Fc (KIH) *F	AML MDS	Phase 1	Aptevo Research and Development LLC	NCT03647800	
CD3×B7-H3	Orlotamab (MGD009)	DART-FC *H	Mesothelioma, Bladder Cancer, Melanoma	Phase 1	MacroGenics	NCT02628535	
	INCA32459-101	Fc-silenced IgG1 *A	Advanced Malignancies	Phase 1	Incyte Corporation	NCT05577182	
CD3×BCMA	Elranatamab (PF-06863135)	IgG2 *A	MM	NA	Pfizer	NCT05565391	
	Linvoseltamab (REGN5458)	IgG4 *A	MM	Available	Regeneron Pharmaceuticals	NCT05164250	
	REGN5459	IgG4 *A	Relapsed/Refractory MM	Phase 1 Phase 2	Regeneron Pharmaceuticals	NCT04083534	
	Pavurutamab (AMG701)	Fab-scFv(bibody) *P	Relapsed/Refractory MM	Phase 1	Amgen	NCT03287908	
	Pacanalotamab (AMG420)	Tandem scFv *K	MM	Phase 1	Amgen	NCT02514239	(138)
	Teclistamab	IgG4 *A	Relapsed/Refractory MM	Marketed	Janssen Research & Development, LLC	NCT05463939	
	EMB-06	Fab-scFv-Fc(KIH) *G	Relapsed/Refractory MM	Phase 1 Phase 2	Shanghai EpimAb Biotherapeutics Co., Ltd.	NCT04735575	
	TNB-383B (ABBV-3830)	IgG4 *A	MM	Phase 1 Phase 2	TeneoOne Inc. AbbVie	NCT03933735	
	ARB202	IgG-HC-scFv *E	Gastrointestinal Cancer Cholangio-carcinoma Liver Cancer	Phase 1	Arbele Pty Ltd Arbele Limited	NCT05411133	
	CC-93269 (EM801)	Fab3CrossMab*J	MM	Phase 1	Celgene	NCT03486067	
	JNJ-64007957	IgG4 *A	Hematological Malignancies	Phase 1	Janssen Research & Development, LLC	NCT03145181	
CD3×CEA	Cibisatamab (CEA-TCB)	Fab3CrossMab*J	Carcinoma, NSCLC	Phase 1 Phase 2	Hoffmann-La Roche	NCT03337698	
	CEA-TCB(RO6958688)	Fab-scFv-Fc(KIH) *G	Solid Tumors	Phase 1	Hoffmann-La Roche	NCT02324257	(139)
CD3×CLEC12A	Tepoditamab (MCLA-117)	IgG1 *A	AML	Phase 1	Merus N.V.	NCT03038230	
CD3×DDL3	TarlatamabAMG757	Fab-scFv (bibody) *P	SCLC	Phase 1	Amgen	NCT04885998	
CD3×EGFR	AMG596	Tandem scFv *K	Glioblastoma, Malignant Glioma	Phase 1	Amgen	NCT03296696	
CD3×EpCAM	Catumaxomab	Rat-mouse hybrid IgG	Solid malignancies	Withdrawn from the market	Neovii Biotech	NCT00836654	
	M701	Fab-scFv-Fc (KIH) *G	MPEs, NSCLC Stage IV	Phase 1 Phase 2	Wuhan YZY Biopharma Co., Ltd.	NCT05543330	
	MT110	Tandem scFv *K	Solid Tumors	Phase 1	Amgen Research Munich GmbH	NCT00635596	
	A-337	scFv2-Fab TriFabs *I	NSCLC	Phase 1	Generon Shanghai Corporation	ACTRN12617001181392	
CD3×FLT3	AMG427	Fab-scFv-Fc (KIH) *G	Relapsed/Refractory AML	Phase 1	Amgen	NCT03541369	
CD3×FcRH5 CD307	Cevostamab	Tandem scFv *K	MM	Phase 1	Genentech, Inc.	NCT03275103	
CD3×GPC3	ERY974	IgG4 *A	Solid Tumors	Phase 1	Chugai Pharmaceutical	NCT02748837	
CD3×GD2	Nivatrotamab (Hu3F8-BSAB)	IgG-scFvLC *D	SCLC	Phase 1 Phase 2	Y-mAbs Therapeutics	NCT04750239	
CD3×GPRC5D	Talquetamab (JNJ-64407564)	IgG4 *A	Hematological Malignancies	Phase 1	Janssen Research & Development, LLC	NCT03399799	
CD3×GPA33	MGD007	DART-FC *H	Colorectal Carcinoma	Phase 1	MacroGenics	NCT02248805	
CD3×gp100	Tebentafusp(IMCgp100)	SCFV-TCR	Uveal Melanoma	Phase 1 Phase 2	Immunocore Ltd	NCT02570308	
CD3×HER2	M802	Fab-scFv-Fc (KIH) *G	HER2-Positive Solid Tumors	Phase 1	Wuhan YZY Biopharma Co., Ltd.	NCT04501770	
	ISB 1302 (GBR1302)	Fab-scFv-Fc (KIH) *G	Breast Cancer	Phase 1 Phase 2	Ichnos Sciences SA Glenmark Pharmaceuticals S.A.	NCT03983395	
	Runimotamab (BTRC4017A)	Undisclosed	Solid Tumors	Phase 1	Genentech, Inc.	NCT03448042	
CD3×HLA-G	JNJ-78306358	IgG4 *A	Neoplasms	Phase 1	Janssen Research & Development, LLC	NCT04991740	
CD3×ROR1	EMB07	Fab-scFv-Fc (KIH) *G	Advanced/Metastatic Solid Tumors	Phase 1	EpimAb Biotherapeutics SuzhouCo., Ltd.	NCT05607498	
CD3×PD-L1	ONO-4685	Undisclosed	Relapsed/Refractory T Cell Lymphoma	Phase 1	Ono Pharmaceutical Co. Ltd	NCT05079282	
CD3×PSMA	AMG160	Fab-scFv-Fc(KIH) *G	NSCLC	Phase 1	Amgen	NCT04822298	
	AMG 340	Tandem scFv *K	mCRPC	Phase 1	Amgen	NCT04740034	
	CC-1	IgG4-SC *A	Lung Cancer Squamous Cell	Phase 1 Phase 2	German Cancer Research Center	NCT04496674	
	ES414	IgG-HC-scFv *E	Prostate Cancer	Phase 1	Aptevo Therapeutics	NCT02262910	
	Pasotuxizumab (BAY20101120)	Tandem scFv *K	Prostatic Neoplasms	Phase 1	Bayer	NCT01723475	
	REGN4336	IgG1 *A	mCRPC	Phase 1 Phase 2	Regeneron Pharmaceuticals	NCT05125016	
CD3×PSCA	GEM3PSCA	Fab-scFv-scFv *P	NSCLC, Prostate Cancer, Renal Cancer, Transitional Cell Carcinoma	Phase 1	AvenCell Europe GmbH GCP-Service International Ltd. & Co. KG	NCT03927573	
CD3×P-cadherin	PF-06671008	DART-FC *H	Neoplasms	Phase 1	Interventional	NCT02659631	(140)
CD3×SSTR2	Tidutamab (XmAb18087)	Fab-scFv-Fc (KIH) *G	Neuroendocrine Tumor, Gastrointestinal Neoplasm	Phase 1	Xencor, Inc.	NCT03411915	
CD27×PD-L1	CDX-527	IgG-HC-scFv *E	NSCLC, Breast Cancer, Gastric Cancer	Phase 1	Celldex Therapeutics	NCT04440943	
CD28×EGFR	REGN7075	IgG1 *A	Advanced Solid Tumors	Phase 1 Phase 2	Regeneron Pharmaceuticals	NCT04626635	(141)
CD39×TGF-β	ES014	IgG-HC-scFv *E	Advanced Solid Tumor	Phase 1	Elpiscience Biopharma, Ltd.	NCT05381935	
MUC2×CD4018	Cemiplimab (REGN4018)	IgG4 *A	Recurrent Ovarian Cancer	Phase 1 Phase 2	Regeneron Pharmaceuticals	NCT03564340	
TCR VB	STAR0602	Fab-scFv-Fc *G	Advanced Solid Tumor	Phase 1 Phase 2	Marengo Therapeutics, Inc.	NCT05592626	
OX40×4-1BB	FS120	IgG1 *A	Advanced Cancer Metastatic Cancer	Phase 1	F-star Therapeutics Limited Merck Sharp & Dohme LLC	NCT04648202	(142)
Targeting immune effector cells: NK cells							
CD30×CD16A	AFM13	Tandem (diabodies) *M	Lymphoma, T-Cell, Cutaneous	Phase 1 Phase 2	Ahmed Sawas Columbia University	NCT03192202	
CD16×CD33	GTB-3550	Tandem scFv *K	HR-MDS AML Systemic Mastocytosis	Phase 1 Phase 2	GT Biopharma, Inc.	NCT03214666	
Targeting immune effector cells: B cells							
CD19×CD64	4G7xH22	IgG-HC-scFv *E	Leukemia Lymphoma	Phase 1	Dartmouth-Hitchcock Medical Center National Cancer Institute NCI	NCT00014560	
CD19×CD22	OXS-1550 (DT2219ARL)	Tandem scFv *K	B-cell lymphoma leukaemia	Phase 1 Phase 2	GT Biopharma	NCT02370160	
CD19×CD47	TG-1801	IgG1 *A	B-Cell Lymphoma	Phase 1	TG Therapeutics, Inc.	NCT03804996	
Targeting multiple checkpoints							
PD-1×CD47	HX009	IgG-HC-scFv *E	Relapsed, Refractory Lymphoma	Phase 1 Phase 2	Waterstone Hanxbio Pty Ltd	NCT05189093	
PD1×CTLA4	Cadonilimab (AK104)	IgG-HC-scFv *E	Advanced Solid Tumors Melanoma	Phase 1 Phase 2	Akeso Pharmaceuticals, Inc.	NCT04172454	
	XmAb20717	Fab-scFv-Fc(KIH) *G	Solid malignancies	Phase 1	Macrogenics	NCT03517488	
	MEDI5752	IgG1 *A	Solid malignancies	Phase 1	AstraZeneca	NCT03530397	
PD-1×ICOS	XmAb23104	Fab-scFv-Fc(KIH) *G	Solid malignancies	Phase 1	Xencor	NCT03752398	
PD-1×LAG3	EMB-02	Fabs-In-Tandem Ig	Advanced Solid Tumors	Phase 1 Phase 2	Shanghai EpimAb Biotherapeutics Co., Ltd.	NCT04618393	
	MGD013	SCFV-Fc(KIH) *F	Advanced Solid Tumors Hematologic Neoplasms Ovarian Cancer	Phase 1	MacroGenics	NCT03219268	
	AK129	IgG1 *A	Advanced Malignant Tumors Stage IA-IB	Phase 1	Akeso	NCT05645276	
PD-1×TIM3	AZD7789	undisclosed	Carcinoma, NSCL	Phase 1 Phase 2	AstraZeneca	NCT04931654	
	Lomvastomig (RO7121661)	IgG1 *A	NSCLC, SCLC, ESCC	Phase 1	Hoffmann-La Roche	NCT03708328	
	LB1410	undisclosed	Solid Tumor Lymphoma	Phase 1	L & L biopharma Co., Ltd., Shanghai China	NCT05357651	
PD1×HER2	IBI315	IgG1 *A	Advanced Solid Tumor	Phase 1	Innovent Biologics Suzhou Co. Ltd.	NCT04162327	(143)
PD-1×VEGF	Ivonescimab (AK112)	IgG-HC-scFv *E	Solid Tumor, Adult	Phase 1 Phase 2	Akeso	NCT04597541	
PD-1×4-1BB	PRS-344/S095012	IgG-HC-scFv *E	Solid Tumor	Phase 1 Phase 2	Pieris Pharmaceuticals, Inc.	NCT05159388	(144)
PD-1×PD-L1	LY3434172	IgG1 *A	Advanced Cancer	Phase 1	Eli Lilly and Company	NCT03936959	
	IBI318	IgG1 *A	Advanced Malignancy	Phase 1	Innovent Biologics Suzhou Co. Ltd.	NCT03875157	
PDL1×4-1BB	MCLA-145	IgG1 *A	Advanced Cancer Solid Tumor, Adult B-cell Lymphoma, Adult	Phase 1	Merus N.V. Incyte Corporation	NCT03922204	
	INBRX-105	SCFV-Fc (KIH) *F	lymphoma, solid tumours	Phase 1	Inhibrx	NCT03809624	
	GEN1046	hetero Fab assembly IgG1 *A	Non-SCLC Metastatic	Phase 2	Genmab BioNTech SE	NCT05117242	
	PM1003	IgG-HC-scFv *E	Advanced Solid Tumors	Phase 1 Phase 2	Biotheus Inc.	NCT05862831	
	ATG101	IgG-HC-scFv *E	Advanced Solid Tumor Metastatic Solid Tumor B-NHLs	Phase 1	Antengene Hangzhou Biologics Co., Ltd.	NCT05490043	
	QLF31907	IgG-HC-scFv *E	Melanoma Urothelial Carcinoma	Phase 2	Qilu Pharmaceutical Co., Ltd.	NCT05823246	
	FS222	IgG1 *A	Advanced Cancer, Metastatic Cancer	Phase 1	F-star Therapeutics Limited	NCT04740424	(145)
	ABL503	IgG-HC-scFv *E	Advanced Solid Tumor	Phase 1	ABL Bio, Inc.	NCT04762641	
PDL1×CTLA4	KN046	IgG1 *A	ESCC, Triple-negative Breast Cancer, Advanced Solid Tumors Lymphoma	Phase 2	Jiangsu Alphamab Biopharmaceuticals Co., Ltd	NCT03925870	
	SI-B003	undisclosed	Solid Tumor	Phase 1	Sichuan Baili Pharmaceutical Co., Ltd. SystImmune Inc.	NCT04606472	
PDL1×LAG3	FS118	IgG1 *A	Advanced Cancer Metastatic Cancer HNSCC	Phase 1 Phase 2	F-star Therapeutics Limited	NCT03440437	
	ABL501	IgG-HC-scFv *E	Advanced Solid Tumor	Phase 1	ABL Bio, Inc.	NCT05101109	
	RO7247669	Fc silenced IgG1 *A	Solid Tumors Metastatic Melanoma NSCLC,ESCC	Phase 1	Hoffmann-La Roche	NCT04140500	
PD-L1×TGF-β	Y101D	IgG-scFvLC *D	Metastatic or Locally Advanced Solid Tumors	Phase 1	Wuhan YZY Biopharma Co., Ltd.	NCT05028556	
	QLS31901	IgG-HC-scFv *E	Advanced Malignant Tumor	Phase 1	Qilu Pharmaceutical Co., Ltd.	NCT04954456	
PDL1×TIGI1	HLX301	IgG-LC-scFv *D	Locally Advanced or Metastatic Solid Tumors NSCLC	Phase 1 Phase 2	Shanghai Henlius Biotech	NCT05102214	
PD-L1×TIM-3	LY3415244	Undisclose	Solid Tumor	Phase 1	Eli Lilly and Company	NCT03752177	(146)
PDL1×VEGF	B1962	IgG1 *A	Neoplasms Malignant	Phase 1	Tasly Biopharmaceuticals Co., Ltd.	NCT05650385	
PD-L1×OX-40	EMB-09	undisclosed	Advanced Solid Tumor	Phase 1	Shanghai EpimAb	NCT05263180	
CTLA-4×LAG3	Bavunalimab (XmAb22841)	IgG-HC-scFv *E	Metastatic Melanoma	Phase 1 Phase 2	University of California, San Francisco	NCT05695898	
CLDN18.2×4-1BB	ABL111TJ0033721	IgG-HC-scFv *E	Solid Tumor	Phase 1	I-Mab Biopharma Co. Ltd.	NCT04900818	
Targeting Growth factors and their recepters							
EGFR×FcγRI	MDX447	IgG-HC-scFv *E	Brain and Central Nervous System Tumors	Phase 1	Dartmouth-Hitchcock Medical Center National Cancer Institute NCI	NCT00005813	
EGFR×MET	JNJ-61186372	IgG1 *A	NSCLC	Phase 1	Janssen R&D	NCT02609776	
	LY3164530	IgG4 *A	Neoplasms	Phase 1	Eli Lilly and Company	NCT02221882	(147)
EGFR×c-Met	MCLA-129	IgG1 *A	NSCLC Metastatic Gastric Cancer ESCC HNSCC	Phase 1 Phase 2	Merus N.V.	NCT04868877	
	EMB-01	Fabs-In-Tandem Ig	Neoplasms Neoplasm Metastasis	Phase 1 Phase 2	Shanghai EpimAb Biotherapeutics Co., Ltd.	NCT05176665	
	Amivantamab	IgG1 *A	NSCLC	Phase 1	Janssen Research & Development, LLC	NCT02609776	
	MCLA-129	IgG1 *A	Solid Tumor NSCLC HNSCC	Phase 1 Phase 2	Betta Pharmaceuticals Co., Ltd.	NCT04930432	
EGFR×4-1BB	HLX35	IgG-HC-scFv *E	Solid Tumors Squamous-cell NSCLC	Phase 1	Shanghai Henlius Biotech	NCT05360381	
EGFR×HER3	SI-B001	IgG-HC-scFv *E	Epithelial Tumor	Phase 1	Sichuan Baili Pharmaceutical Co., Ltd. SystImmune Inc.	NCT04603287	
	Duligotuzumab (MEHD7945A)	IgG1 *A	Neoplasms	Phase 1	Genentech, Inc.	NCT01986166	
EGFR-LGR5	Petosemtamab(MCLA-158)	IgG1 *A	Solid Tumors NSCLC HNSCC	Phase 1 Phase 2	Merus N.V.	NCT03526835	
HER2×HER2	Alphamab (KN026)	IgG1 *A	breast and gastric cancer	Phase 1	Jiangsu Alphamab Biopharmaceuticals Co., Ltd	NCT03619681	
	MBS301	IgG1 *A	Solid malignancies	Phase 1	Beijing Mabworks Biotech Co., Ltd.	NCT03842085	
	ZW49	Fab-scFv-Fc (KIH) *G	HER2-expressing Cancers	Phase 1	Zymeworks Inc.	NCT03821233	
ECD2×ECD4HER2	ZW25	Fab-scFv-Fc (KIH) *G	HER2-Positive Advanced BTC	Available	Jazz Pharmaceuticals	NCT04578444	
HER2×HER3	MM-111	IgG-scFv *	Her2 Amplified Solid Tumors Metastatic Breast Cancer	Phase 1	Merrimack Pharmaceuticals	NCT00911898	
	Zenocutuzumab (MCLA-128)	IgG1 *A	Tumours Harboring NRG1 Fusion	Phase 2	Merus N.V.	NCT02912949	(148)
	Zenocutuzumab (MCLA-128, PB4188)	IgG1 *A	breast cancer	Phase 2	Merus	NCT03321981	
HER2×4-1BB	YH32367 (ABL105)	IgG-HC-scFv *E	HER2-Positive Solid Tumor	Phase 1 Phase 2	Yuhan Corporation	NCT05523947	
IGF1R×HER3	Istiratumab (MM-141)	IgG1 *A -scFv *E	Colorectal Cancer NSCLC HNSCC	Phase 1	Merrimack Pharmaceuticals	NCT02538627	
VEGF×Ang2	BI 836880	Tandem VHH *O	NSCLC	Phase 1	Ablynx/ Boehringer Ingelheim	NCT02689505	
	Vanucizumab, (RO5520985)	IgG1 *A	Solid malignancies	Phase 1	Roche	NCT02715531	
VEGF×DLL4	Dilpacimab (ABT-165)	Tandem Fv-IgG1 *A	CRC	Phase 1	AbbVie	NCT03368859	
	Navicixizumab	IgG2 *A	ovarian, peritoneal fallopian tube cancers	Phase 1	Celgene/Oncomed	NCT03030287	
	NOV1501 (ABL001)	IgG-HC-scFv *E	Advanced Solid Tumors	Phase 1	ABL Bio, Inc. National OncoVenture	NCT03292783	
Targeting other points							
CD73×TGFβ-Trap	Dalutrafusp alfa (AGEN1423)	IgG1 *A	PDAC	Phase 2	Bruno Bockorny, MD Agenus Inc.	NCT05632328	
CD40×MSLN	ABBV-428	SCFV-Fc (KIH) *F	Solid malignancies	Phase 1	AbbVie	NCT02955251	
CEA×HSG	CrossMabTF2	Tandem scFv *K	SCLC CEA-expressing NSCLC	Phase 1 Phase 2	Centre René Gauducheau	NCT01221675	

Data available as of 1 August 2023. Molecules are ordered on the basis of the antigens in the first column. The capital letters after * in the third column represent the type of BsAbs in **Figure 2**. Fab, antigen-binding fragment; ScFv, single-chain variable fragment; DLBCL, diffuse large B-cell lymphoma; MCL, mantle cell lymphoma; B-ALL, B cell acute lymphoblastic leukaemia; IgG, immunoglobulin G; Fc, fragment crystallizable region; VHH, variable heavy-chain only fragment antibodie; BCL, B-cell lymphoma; FL, follicular lymphoma; NHL, non-hodgkins lymphoma; CLL, chronic lymphocytic leukemia; LBCL, large B-cell lymphoma; SCT, stem cell transplantation; AML, acute myeloid leukemia; LUSC, lung squamous cell carcinoma; MDS, myelodysplastic syndromes; MM, multiple myeloma; 7-H3, B7 homologue 3; BCMA, B cell maturation antigen; CEA, carcinoembryonic antigen; CLEC12A, C-type lectin domain family 12 member A; DLL3, delta-like ligand 3; EpCAM, epithelial cell adhesion molecule; FLT3, Fms-like tyrosine kinase 3; FcRH5, Fc receptor homologue 5; GPC3, glypican 3; GPRC5D, G protein-coupled receptor family C group 5 member D; GPA33, Glycoprotein A33; gp100, glycoprotein 100; HER2, human epidermal growth factor receptor 2; HLA-G, human leucocyte antigen-G; ROR1, receptor tyrosine kinase-like orphan receptor 1; PD-1, programmed cell death 1; PD-L1, programmed cell death 1 ligand 1; SSTR2, somatostatin receptor 2; EGFR, epidermal growth factor receptor; TGF-β, transforming growth factor-β; MUC2, recombinant mucin 2; TCR, VBT-Cell receptor; OX40, tumor necrosis factor receptor superfamily member 4; 4-1BB, tumor necrosis factor receptor superfamily member 9; CTLA4, cytotoxic T lymphocyte antigen 4; PSMA, prostate-specific membrane antigen; ICOS, inducible T cell co-stimulator; LAG3, lymphocyte-activation gene 3; TIM3, T cell immunoglobulin mucin 3; VEGF, vascular endothelial growth factor; CLDN18.2, Claudin18.2; FcγRI, receptor I for the Fc region of immunoglobulin G; MET, mesenchymalepithelial transition factor; c-MET, cellular-mesenchymalepithelial transition factor; HER3, human epidermal growth factor receptor 3; LGR5, leucine-rich repeat-containing G protein-coupled receptor 5; IGF1R, insulin-like growth factor 1; Ang2, Angiopoietins2; DLL4, delta-like ligand 4; MSLN, mesothelin; HSG, hysterosalpingography; NSCLC, non-small cell lung cancer; SCLC, small cell lung cancer; MPEs, malignant pleural effusions; mCRPC, metastatic castration-resistant prostate cancer; ESCC, esophageal squamous cell carcinoma; B-NHLs, mature B-cell non-hodgkin lymphoma; HNSCC, head and neck squamous cell carcinoma; HR-MDS, high-risk myelodysplastic syndromes; BTC, biliary tract cancer; PDAC, pancreatic ductal adenocarcinoma; NK, natural killer.

### 5.1 Reorientating immune effector cells

**Figure 5:**
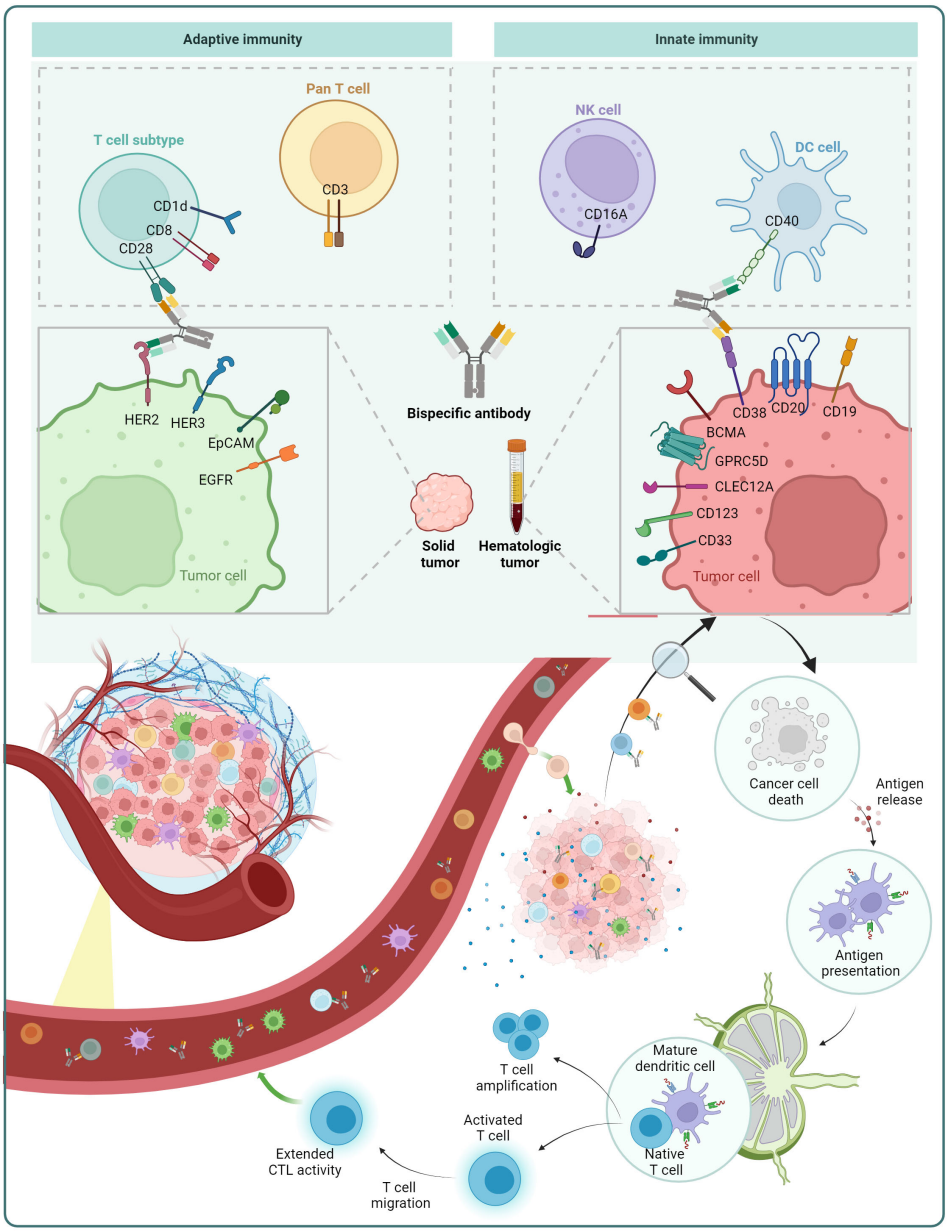
Mechanism of action of BsAb: redirection of immune cells. BsAb can redirect pan T cells and T cell subsets from the adaptive immune system, as well as natural killer (NK) cells and dendritic cells (DCs) in the innate immune system. The figure also displays some of the immune cell surface antigens and tumor cell surface antigens that are already under investigation.

In the case of cancer treatment, one arm of the BsAb targets a tumor-associated antigen (TAA), while the other arm targets a molecule present on immune cells. Via targeting both the TAA on tumor cells and immune cell molecules, the BsAb brings the immune cell in close proximity to the tumor cell, resulting in the immune cell’s stimulation and then destroying the tumor cell (**Figure 5**).

Under microhomeostatic circumstances, anticancer immunity is one of the essential cancer therapy techniques. However, in order to survive and propagate, tumor cells are able to escape the “cancer-immunity cycle” which describes how the innate and adaptive immune systems collaborate to prevent malignancy genesis through sequential events (149). This is accomplished by mechanisms that suppress anti-tumor immunity, such as increased expression of molecules like PD-L1 that block T-cell action or decreased production of human leucocyte antigen (HLA) class I molecules that hinder antigen presentation (150–153).

Substantial therapy outcomes can be attained by reviving and strengthening the latent immune cells, which has been demonstrated by the development of several mAb immune checkpoint inhibitors (ICIs) that bind PD1, PD-L1, CTLA-4, etc. during the past ten years (154–157). Major improvements in total and advancement-free survival have been obtained in melanoma, lung carcinoma, and urothelial cancer (58, 130).

#### 5.1.1 Reorientating the cells of the adaptative immune system

As of October 2023, the majority of BsAbs bridge cells as their primary action mode and have T cell reorientation as their shared thread. Rerouting effector T cells with cytotoxic activity to destroy malignant cells is a classic paradigm of these BsAbs (28).

BiTEs primarily stimulate T cells via interaction with CD3ϵ in the T-cell receptor (TCR) complex, thus are defined as pan-T-cell engagers (158, 159). The high affinity between BiTEs and TAA/CD3a renders a huge proportion of activation receptors (TCR/CD3 complexes) gather between cells, resulting in effectual T-cell excitation with just one receptor-ligand interplay and the killing of cancer cells through releasing perforin and granzymes (130, 160, 161). Most CD3-targeted pan-T-cell stimulators at the clinical phase are designed to treat blood tumors, such as targeting CD19 and CD20 for non-Hodgkin lymphoma (NHL), targeting B cell maturation antigen (BCMA), GPRC5D and CD38 for multiple myeloma (MM), targeting CD33, CD123, and CLEC12A for acute myeloid leukemia (AML) (162). Although these targets are widely expressed on healthy blood cells as well, their depletion can be handled without eliciting serious negative impacts (114). A relatively small amount of TCRs target MHC-presented TAAs in solid tumors (163). Phase I clinical studies are being conducted with AMV564, a TandAb with two CD3 binding sites and two CD33 binding sites respectively (162).

The TAA selection is crucial for BiTEs to perform properly. The performance of BiTEs is associated with expression ratios of targets, as was the case with BiTEs targeting EpCAM, CD33, and HER2 (164). Three distinct cell lines exhibiting high (EOL-1), medium (MOLM-13), and low (MV4-11) rates of FMS-like tyrosine kinase 3 (FLT3) expression were employed to analyze the influence of receptor density on the potency of FLT3 BsAb *in vivo*. Compared with the MOLM-13 model, the EOL-1 model demonstrated total potency at a lower dose of 7370, which is aligned with EOL-1’s higher membrane FLT3 expression (165). BiTEs’ activity is also influenced by TAA’s characteristics, such as size and mobility on cell membrane (166). Chinese hamster ovary (CHO) cells presenting small surface target antigens were typically more effectively lysed than those with bigger antigens (167). The antigen’s affinity to candidates is also an important determinant for the strength of BiTE. High affinity for HER2 was essential for the HER2/CD3-targeted BiTEs’ ability to destroy cancer cells. Nevertheless, a worse safety profile, such as cytokine release and impairment to HER2-expressing tissues, was also linked to increased HER2 affinity. Adopting a dose-fractionation method could enhance the HER2/CD3-targeted tolerance (168).

Architecture of CD3-binding part impacts the biodistribution of BiTEs. Despite BiTEs with high CD3 affinity demonstrated better efficacy in co-culture tests *in vitro*, a reduced affinity of the CD3-binding domain is preferred to enable effective tumor diffusion *in vivo* without triggering rapid CD3-regulated plasma elimination or antibody entrapment in organs which store T cells (169–173). Many BiTEs only have one CD3-binding domain, whereas some clinical-stage BsAbs possess two CD3-targeting sites. However, whether such formats could functionally connect CD3 bivalently is unclear, which is essential for antigenic regulation and tolerance evoked by CD3-mAbs (28, 174). A monovalent CD3 interplay is favored because bivalent CD3 binding may crosslink the TCR/CD3 complex even if it is not simultaneously bound to TAA-expressing cells, resulting in systemic T-cell stimulation and cytokine release syndrome (CRS) (114, 175).

There are several FDA-authorized BiTEs for tumor therapy. In 2009, catumaxomab was granted clinical approval as the first BiTE. This antibody has two distinct antigen-targeting sites—one for the CD3 antigen on T-cells and another for the EpCAM on tumor cells—and also binds to accessory cell FcγR via its preserved Fc region (9). However, the IV injection of catumaxomab was linked to serious harmful effects that were ascribed to the active Fc site’s off-target adhesion to other immune cells expressing FcγRs, causing CRS and T cell-mediated hepatic damage (114, 176, 177). In 2014, Blinatumomab, a BiTE created by Amgen Inc. for the treating blood malignancies derived from B-cell lines (178), was authorized by the FDA for the therapy of acute lymphoblastic leukemia (ALL). Blinatumomab is a tiny BsAb with a molecular weight of around 55 kDa and a brief plasma half-life of 1.25±0.63 hours *in vivo *(28, 179–181). In this regard, the switch from sporadic IV infusion to constant IV infusion was a crucial choice in the clinical development of blinatumomab, which not only elevated security but also permitted more sustained T cell activity by preserving effective drug serum rates for the duration of a treatment cycle (182). Motivated by the promising clinical data of binatumomab, a variety of BiTEs with multivalent TAA affinity and monovalent CD3 binding, as well as the DART format have been developed to improve tumor selectivity and reduce off-target side effects (28, 114). In 2021, zenocutuzumab, an innovative IgG1 class HER2/HER3 BsAb utilizing the “dock-and-block” strategy, was granted the Fast Track Designation for NRG1^+^ metastatic neoplasms. Owing to its selectivity for HER2’s domain 1, zenocutuzumab can suppress HER2/HER3 signals regardless of the presence of HER2. Furthermore, it has no synergistic toxicity on cardiac myocytes conducted by HER2/HER4, thanks to its selectivity in blocking HER2/HER3 dimerization (183, 184).

Despite their potential, certain investigations revealed that T cells activated by BiTEs become less potent over time since they deplete more quickly (130, 185). As is the case with blinatumomab and catumaxomab, the administration of BiTEs is linked to CRS, which is indicated by abrupt elevations in the serum amounts of inflammatory cytokines such interleukins-6 (IL-6), tumour necrosis factor (TNF), and interferons (IFNs) (186–188). In an intriguing study applying an anti-PSMA T-BsAb, the scFv-Fc-scFv T-BsAb design allowed for the generation of powerful T-cell-dependent cellular cytotoxicity (TDCC) *in vitro* while limiting cytokine release, indicating that carcinoma cytotoxicity and cytokine storm might be distinct events or that an ideal balance between efficacy and toxicity can be realized by altering BiTE layout (189). Another significant drawback of CD3-targeted BiTEs is a significant fraction of the T-cell population is awakened. Therefore, compared to existing CD3-targeted pan-T-cell activators, BiTEs specifically activating distinct T-cell subtypes would be advantageous (114).

Targeting particular T cell subgroups render BsAb more effective in the destruction of tumor cells (28). Blinatumomab could stimulate Tregs *in vitro*, which inhibited effector T cells’ cytotoxicity (190). Additionally, in 42 patients with B cell ALL, the number of Tregs in the peripheral blood before blinatumomab administration negatively predicted response (190). Therefore, one of the objectives to construct a CD8^+^ T cell and prostate stem cell antigen binding tandem scFv was to avoid the induction of Tregs (40, 191). Vγ9Vδ2-T cells are a small and conserved T-cell fraction with a powerful inherent immunotherapeutic prospective. Vγ9Vδ2-T cell concentration and powerful CD1d-reliant tumor lysis are made possible by a bispecific Vγ9Vδ2-T cell engager (192).

Obstructing inhibitory checkpoints can revive weary neoplasm-permeating T cells (114). Inhibitory receptors such as PD1, mucin­domain containing­3 (TIM3), and lymphocyte­activation gene 3 (LAG3) are abundantly expressed when T cells are in a worn-out condition, which results in defective effector outcomes (193–195). Immune checkpoint-targeting BsAbs are arising following the therapeutic efficacy of anti-CTLA4 (cytotoxic T lymphocyte antigen 4), anti-programmed cell death protein 1 (PD-1), and anti-PD-L1 antibodies, while the enhanced therapeutic effect seen in coupled research using mAbs that engage the checkpoints serves as justification for concurrently engaging two immune checkpoints (28, 196). The majority of the BsAbs inhibit the PD-1/PD-L1 pathway with one arm while blocking CTLA-4, LAG3, or TIM3 with the other (28, 114). Fc-silenced BsAbs were developed to block the PD-1 cascade via high-affinity PD-1 interaction whilst obstructing CTLA4 with a low-affinity binding domain in order to enhance the safety aspect of simultaneous engagement of PD-1 and CTLA4 (28).

#### 5.1.2 Reorientate cells of the innate immune system

BsAbs are also evolving as a substitute therapy strategy geared at the induction of intrinsic immune effector cell toxicity versus cancers with prospects for therapeutic potency and reduced therapeutic toxicity (197, 198). The bulk of BsAbs regarding the innate immune system focuses on dendritic cell (DCs), natural killer (NK) cells, and phagocytes (114, 199).

DCs are professional antigen-presenting cells (APCs). BsAbs with intact Fc domain can be employed to boost the chances of DCs and T cells coming into contact (130). In this regard, a BsAb which concurrently and agonistically activated CD28 on naïve T cells and CD40 on AML-DC was developed. In addition to improving CD28-mediated messaging, it was proposed that the ensuing cellular cross-linking would strengthen and prolong T cell/AML-DC contacts, thus boosting T cells’ sensitivity to AML antigens (200).

NKs may identify and destroy stressed cells, triggering an immune response much more quickly without antibodies or MHC. Tandem scFv, also known as “bispecific killer cell engager” (BiKE) or “trispecific killer cell engager” (TriKE), is a technique for directing NK cells toward cancer cells (201, 202). The innate cell engager (ICE^®^) AFM13 is a tetravalent BsAb that targets CD16A, the main FcR on NK cells, and CD30, which is prevalent in blood malignancies. In individuals with recurrent or resistant Hodgkin lymphoma, it has exhibited early clinical efficacy without significant therapeutical toxicity (197, 203–207). NK cells can also be recruited to cancer cells based on a scFv-Fc-scFv format. RO7297089, a bispecific BCMA/CD16A-targeted ICE^®^ intended to cause BCMA^+^ MM cell lysis via strong affinity interaction of CD16A and redirection of NK cell toxicity and macrophage phagocytosis, promotes antibody-dependent cell-mediated cytotoxicity (ADCC) and ADCP against myeloma cells effectively, as well as pharmacodynamic efficacy in cynomolgus monkeys (197).

The modification of *in vitro* activated or expanded immune cells with BsAbs represents a new therapy for cancer treatment. The first clinical report of this approach emerged in 1990. Nitta et al. applied the method to malignant gliomas by using anti-CD3/glioma BsAb-modified lymphokine-activated killer (LAK) cells, which exhibited a favorable anti-tumour effect (208). Following the emergence of cytokine-induced killer (CIK) technology, modified CIK cells with BsAbs have been introduced into clinical studies (209). In nude mice, the use of BsAb-CIK cells resulted in a significant (p<0.05) reduction in CD133 (high) tumour growth (210). Golay et al. utilized CIK cells from cryopreserved cord blood units along with blinatumomab for the treatment of CD19 malignancies, which showed meaningful therapeutic effects with no evidence of toxicity or graft-versus-host disease (210). BsAbs *ex vivo* armed T cells (EAT) could potentially overcome certain limitations of chimeric antigen receptor-modified T cells (CAR-T), including cytokine release syndrome and neurotoxicity (211). Park et al. developed EAT utilizing the IgG-[L]-scFv platform BsAb. This resulted in a more efficient infiltration of tumors and a lower concentration of TNF-α released compared to the use of BsAb or T-cell injections alone (212).

### 5.2 Delivery of medicines

The distribution of payloads such as medicines, radiolabels, and nanoparticles is an intriguing deployment of BsAbs (97). A payload comprising an isotope or a medication is directly attached to a BsAb in this method (40).

#### 5.2.1 Radioactive payload

**Figure 6:**
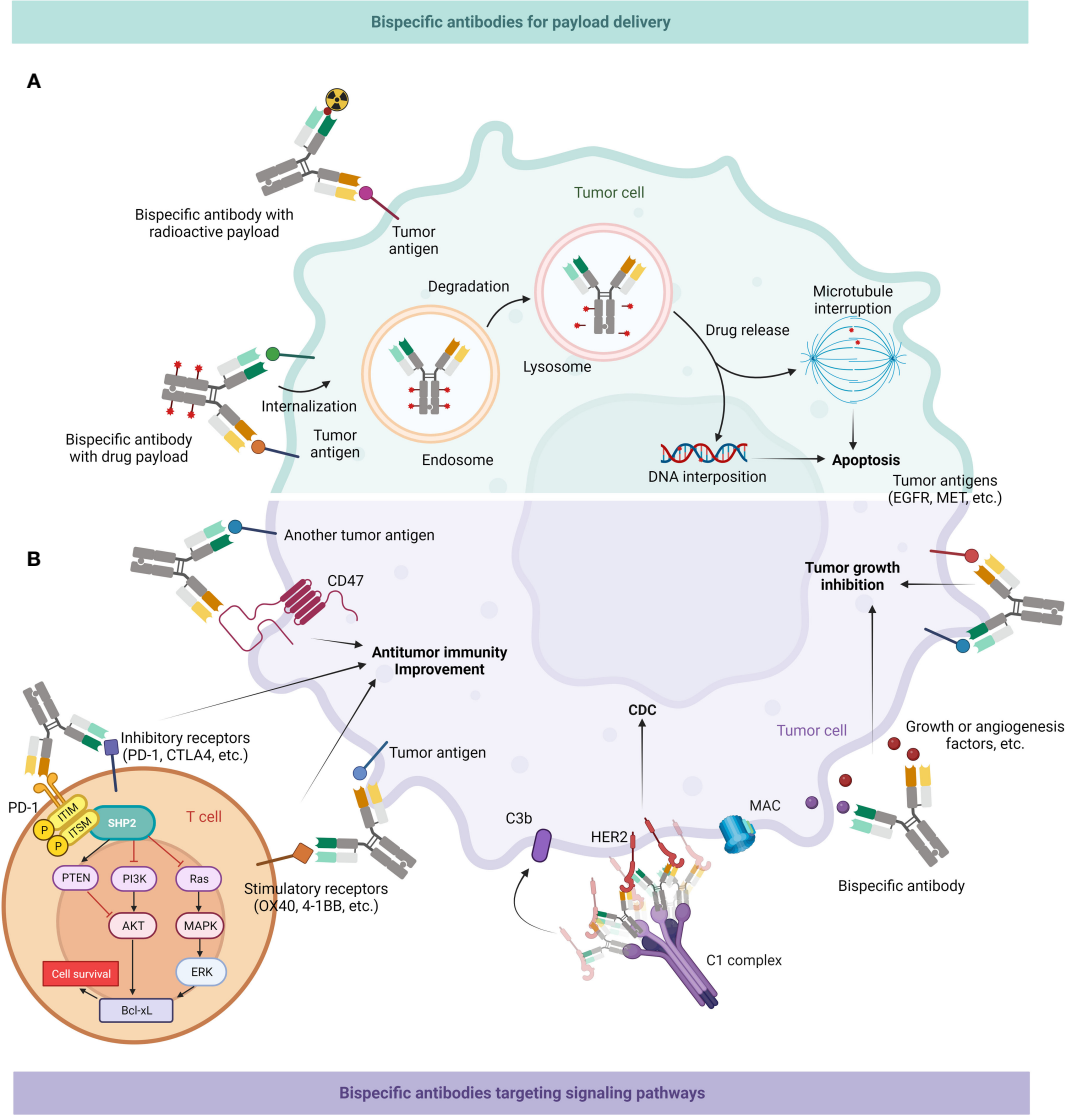
Mechanism of action of BsAb: delivery of payloads & targeting tumor-associated signaling pathways. **(A)** BsAb can enrich radioactive substances around tumor cells; they might also load drugs and then enter cancer cells through endocytosis, first into endosomes and then into lysosomes, and then release drugs to interfere with microtubule formation or DNA replication, ultimately leading to apoptosis of tumor cells. **(B)** BsAb can directly inhibit tumor growth. It can interfere with multiple signaling pathways related to neoplastic growth, as well as inhibit growth factors or angiogenic factors in the tumor microenvironment (TME). BsAb may also facilitate antitumor immune activity. It can bind CD47 on the surface of tumor cells to promote macrophages-mediated phagocytosis of them, or target inhibitory or stimulatory receptors on T cell surfaces to enhance the effector function of T cells. BsAb can also form hexamers by binding to receptors like HER2, recruiting the C1 complex, and subsequently mediating tumor cell death through the CDC effect (216).

Inferior therapeutic indices (TI) cause the majority of radioligand treatments to have unforeseen dose-limiting toxicities to crucial organs, which leads to many patients receiving subtherapeutic dosage of treatment. BsAbs can be applied to enhance radioactive payloads in neoplasm areas, which can greatly increase the tumor/blood percentage and serum retention duration (97, 213–215) (**Figure 6A**).

A BsAb with specificity for both the TAA and the load can be cultured with the load before infusion (40). Optionally, pre-targeting strategies can be applied to avoid sustained exposure of normal tissue to the payload, reducing toxicity and side effects, which involve first injecting the BsAb, followed by administering the payload to deliver radioactive loads to a malignancy (40, 217). Such BsAbs with a radioactive payload could be utilized for radioimmunotherapy as well as cancer imaging. Pilot research revealed that pretargeted immunological positron emission tomography (immuno-PET) employing a CEA/IMP288-targeted BsAb and a [^68^Ga] Ga-labelled hapten was secure and practical with encouraging diagnostic accuracy (218). A creative cancer-targeted DOTA-hapten pre-targeted radioimmunotherapy platform, Pr, is composed of a vacant DOTA-chelate for ^225^Ac, which is connected to a lutetium-composited DOTA for picomolar DOTA-bound chelate scFv adhering through a short polyethylene glycol linker. In three solid patient tumor xenograft models for neuroblastoma (GD2), colorectal cancer (GPA33), and breast cancer (HER2), extended general survival, complete responses, and histologic healing were noted. Also, [^225^Ac]Pr has a significantly higher security profile in contrast with RIT with tumor-targeted IgG antibodies (219).

#### 5.2.2 Drug payload

By combining targeted treatment with a strong cytotoxic payload to antibodies, antibody-drug conjugate (ADC) medicines destroy malignant cells through a pharmaceutical Trojan horse approach (37) (**Figure 6A**). The monoclonal antibody-based ADCs, including the anti-CD33 drug gemtuzumab ozogamicin, the first ADC authorized by the FDA, and the anti-HER2 drug adotrastuzumab emtansine to treat advanced breast carcinoma, have achieved a significant progress (220).

However, the potency of therapy may be restricted gradually since tumors may evolve defences against drug effects. Downregulation of the antigen on the cell membrane, which makes the ADC comparatively less likely to perform the cytotoxic activity, may be one cause of resistance (221, 222). Bispecific or biparatopic mAbs, which bind to non-overlapping epitopes on a single target antigen or two distinct antigens simultaneously, are novel strategies to circumvent antigen-specific rejection mechanisms (223). M1231 is an experimental ADC combining a BsAb which concurrently targets MUC1 and EGFR with a payload linked to hemiasterlin. Patients with progressive solid tumors, such as esophageal cancer, and non-small-cell carcinoma (NSCLC) are currently being studied using the BsAb as a monotherapy in phase I trials (224).

It is vital to internalize proteins effectively and direct them to lysosomes where proteolysis can occur. Nevertheless, the amplitude of these activities for many cell membrane proteins and carbohydrate compounds on tumor cells is inadequate to support an efficient ADC strategy. One approach is to incorporate BsAbs into ADCs, where one binding region would offer malignant affinity while the other binding site would enable localization to the lysosome (224). A bispecific ADC that targets both HER2 and the lysosome membrane protein CD63 appears to increase lysosomal aggregation as well as cargo delivery (225, 226).

#### 5.2.3 Targeted delivery of nanoparticles

Delivery of nanoparticles with BsAbs can overcome the inefficiency of the enhanced permeability and retention (EPR) effect of nanomaterials and improve the active targeting and delivery efficiency of the drug itself. At the same time, BsAbs can enhance the anti-tumour activity of nanoparticles and reduce the amount of drug used, thus reducing off-target cytotoxicity.

Active targeting of nanomaterials can be achieved through coupling BsAbs to the surface of the nanomaterials. BsAbs and nanoparticles are linked via antibody-antigen interactions, which are more specific than random coupling methods (227). The vast majority of connections between BsAbs and nanoparticles are non-covalent, and upon cellular endocytosis of drugs, these bonds are readily broken, thus increasing drug release and improving delivery efficiency (228). Furthermore, the selectivity of BsAbs allows for the specific targeting of tumour tissues through nanomedicines, holding immense significance in the treatment of solid tumors. EGFR-targeted EDV nanocells loaded with paclitaxel are currently part of clinical studies and can be safely administered to patients with advanced solid tumors at a maximum tolerated dose (MTD) of 1×10^10^ microcells per dosage (229). Pre-targeted therapies are another method of delivering nanomedicines based on BsAbs (230). A CD20 Ab-mPEG scFv was able to target CD20-expressing Raji cells while carrying simultaneously grasping polyethylene glycolated liposomal DiD with a 24% increase in internalization capacity (60 h). It showed a nine-fold increase in tumour cytotoxicity (LC50: 3.45 nM) and improved anti-liquid tumour efficacy (p=0.005) compared to CD20 Ab-mPEG scFv and PLD alone (231). Another study showed that BsAbs pre-targeted delivery of D-Dox-PGA reduced off-target toxicity in human breast cancer xenografts and had antitumor efficacy comparable to Dox therapy (232).

### 5.3 Signaling pathway targeting

Some cancer cells can develop resistance to single-target therapies by activating alternative signaling pathways. BsAbs can co-target several tumor-related receptors on the cell membrane (**Figure 6B**), providing powerful anticancer strategy and reversing resistance, eliciting a more potent and effective response than single-target antibodies.

#### 5.3.1 Bridge receptors

Resistance is a main downside of RTKs, such as EGFR and HER2. Resistance typically entails the activation of parallel signal transduction by upregulating other RTKs that avoid targeted receptor blockage. For example, the MET oncogene is amplified to induce HER3 and the PI3K-AKT cell survival pathway, enabling NSCLC to escape the suppression of EGFR-TKIs (226, 233). Several BsAbs co-targeting several RTKs were developed and are currently undergoing clinical trials (233).

The members of the ErbB family, EGFR, HER2, and HER3, are typical targets for BsAbs that interrupt two signals because of their interaction (40, 234–236). Zenocutuzumab targets the HER2 and HER3 cytoplasmic regions. By blocking Neuregulin 1 (NRG1) binding to HER3 and preventing HER3 from going through the conformational alteration necessary for heterodimerization with HER2 and possibly with EGFR, zenocutuzumab inhibit downstream oncogenic signals and the phosphorylation of HER3’s cytoplasmic region (148, 183). Clinical success was verified in patients with pancreatic and lung malignancies induced by NRG1 rearrangements (148). Other oncogenes such as MET can also become targets. Amivantamab (JNJ-372) is a first-in-class, completely humanized, IgG1 BsAb that targets both EGFR and MET synchronically. It hinders the stimulation of ligand-binding receptors, encourages their internalization and destruction to impede downstream signaling cascades. It can involve Fc-mediated attachment to macrophages and then trigger a potent antibody-reliant ADCC which is vital for the blockage of both EGFR and MET pathways (38, 39, 237). Additional targets under research include lysosomal internalization-related receptors like CD63 and death receptors like death receptor 5 (DR5) (40). Shivange et al. develop antibodies that limit DR5-mediated apoptotic activity toward FOLR1-expressing ovarian tumor cells yet minimizing the need for ADCC to trigger cell destruction. It turned out that these antibodies operated better than DR5 agonist antibodies reported from clinical testing (238).

Besides, the activation of the complement-dependent cytotoxicity (CDC) system is regarded as a method for enhancing the clinical efficiency of anticancer Abs (239). It comprises one of the identified modes of action (MOA) for B-cell specific monoclonal Abs such as rituximab and atumumab (240–242). The capacity of the antigen-Ab combination to adopt a shape which permits effective C1q interaction is influenced by an array of variables, such as antigen diameter and density, which regulate activation of the classical complement system (243). Hexameric arrangement of Ab Fc regions in the Ab-antigen complexes is necessary for optimum CDC action (244). In preclinical investigations, techniques that improve CDC, such as Ab hexamerization and Fc alterations, have showed potential for improving anticancer efficacy. For instance, hexamerization was employed to create a biparatopic anti-CD37 B-cell specific Ab with increased *in vitro* CDC efficacy (216, 245). Utilizing BsAbs which antagonize the C-mediators CD55 and CD59 to promote C-regulated activities, Macor et al. devised an innovative method to improve CDC (246). Although trastuzumab, pertuzumab, and tras+pert exert anticancer action via a variety of MOAs, they are unable to induce CDC in HER2-exhibiting cells when human serum is present (247–249). According to Weisser et al.’s hypothesis, a biparatopic Ab designed to increase receptor crosslinking and gathering might offer the receptor-Ab complex needed to connect C1q and activate the effective anti-cancer mechanism, as well as preserve and/or improve all other identifiable anti-cancer MOA assigned to authorized anti-HER2 Ab therapies (216).

#### 5.3.2 Biparatopic BsAbs

BsAbs might be constructed to concurrently attach to two non-overlapping epitopes on the same target rather than two distinct antigens. Biparatopic targeting mimics the actions of antibody cocktails and polyclonal antibodies by enhancing binding efficiency via antigen crosslinking and accumulation (28). Earlier research has demonstrated that it is crucial for biparatopic BsAb to structurally identify two epitopes or to have a strong specificity for the antigen (250). Therefore, the membrane protein, which has extensive extracellular domains and has been the subject of a few mAbs, is a viable target for BsAbs (251). ZW25 is a BsAb that adheres to two HER2 epitopes concurrently, one is the pertuzumab (Perjeta; Genentech) binding site ECD2, and one is the trastuzumab binding site ECD4. According to preclinical studies, ZW25 are able to mute HER2 signaling more efficiently than trastuzumab or pertuzumab and exhibit high anticancer efficacy at a variety of HER2 expression rates. Currently, the drug is being evaluated in a phase I basket test for HER2-positive tumors (11).

#### 5.3.3 BsAb targeting redundant ligand

Another area of concern for BsAbs is tackling redundancy for numerous growth or angiogenesis factors in the tumor microenvironment (TME). By reducing angiogenin-2 (Ang-2) and vascular endothelial growth factor-A (VEGF-A) in the TME, the CrossMab construction vanucizumab prevents angiogenesis, while VEGF and delta-like ligand 4 are the objectives of the BsAb OMP-305B83. In these architectures, both BsAbs carry Fc as a lengthy half-life is essential for efficient factor elimination (40). Moreover, ZVEGFR2 Bp2, which has anti-angiogenic impacts equivalent to the clinically authorized Ramucirumab, was regarded as the best candidate for further assessment because of the slow dissociation level and the benefits of a shorter linker in a study to test the potential of four biparatopic constructs (252).

## 6 Challenges and prospects in solid tumor

The application of BsAbs in cancer has also encountered numerous bottlenecks, particularly in solid tumors, which are mainly attributed to the more complex microenvironment of solid tumors. In this section, we will discuss the challenges of identifying suitable targets within solid tumors, penetrating the tumor, as well as the difficulties related to tolerance, side effects, and large-scale production of BsAb therapy.

### 6.1 Identifying the promising targets in solid tumor

For solid malignancies, the first step to effective immunotherapy is identifying the right targets, which are probably TSAs uniformly expressed on cancer cells and are crucial in the growth of tumors (253). Although both immune cell-redirecting and antigen-bridging BsAbs are being investigated, over three-quarters of these investigations focus on the second one. Bridged antigen pairings can be single targets like HER2/HER2 (biparatopic BsAb) or associations of two separate antigens like PD-1/CTLA4, PD-1/PD-L1, VEGF/Ang-2, IGF-1/IGF-2, etc. These BsAbs primarily target double signaling transductions to produce reinforced inhibited or activated impacts in lymphocytes or tumor cells. Immune cell-redirecting, CD3-targeted/TAA-targeted BsAbs have used TAAs such as EGFR, HER2, and c-Met, whereas TSAs like EGFRvIII (254), p95HER2 (255), and RAS G12V are more desirable targets to prevent on-target, off-tumor impacts caused by target expression on healthy cells. In fact, hemopoietic differentiation antigens like BCMA, CD123, and CD20 satisfy these requirements, while there are few TSAs accessible for solid tumors and, if exists, antigen heterogeneity would be an issue (256).

### 6.2 Penetration of the immune cells

Only a modest percentage (less than 1%) of migrated T cells were ultimately able to penetrate solid neoplasm tissues, according to preclinical research (257). Therefore, in order to maximize the efficacy of immunotherapies in cancers, T cell penetration must be improved (258). Macromolecules like BiTEs force out via transvascular holes with diameters ranging from 200 nm to 1.2 μm inside the solid tumor vasculature and permeate into the neoplasm (259). TILs are present in a variety of solid malignancies, and their number is closely correlated with cancer reaction to ICIs and prognosis (260–262). In malignancies lacking TILs (263), it is depended on the BiTEs’ capacity to draw T cells from the blood. The TME presents a variety of difficulties for BiTE medication, as it does for other macromolecule treatments for solid malignancies. A solid tumor’s physical hurdle prevents trafficking and penetration, greatly lowering the quantity of both BiTEs and T cells involved (264). An alluring remedy is the gene editing of an oncolytic virus to generate a BsAb following its invasion of a tumor cell. After selectively infecting tumor cells, the virus releases its offspring into the surroundings. In the vicinity of the tumour, the virus releases BiTEs that trigger a T cell activation during the viral cycle. A study reveals the viability and efficiency of using an erythropoietin­producing human hepatocellular carcinoma A2 receptor (EpHA2)-targeted BiTE in combination with oncolytic adenoviruses and adenovirus-delivered gene treatment; this design can provide a robust tumor-specific cytotoxic response that brings long-term management of pediatric high-grade gliomas (265). Adenoviruses and other oncolytic viruses can also destroy cells immediately via oncolysis, which might cause the liberation of neoantigens from the ruptured cells. The APCs’ further exposure of them may serve as an *in situ* vaccine, boosting the precise immune reaction (266). Comparable methods are reported by Gardell et al., who administered a retrovirally edited macrophage that could release a BiTE selective for EGFRvIII to mouse models of glioma. The macrophages would stay in the solid malignancy and continue to secrete IL-12 and BiTE, which would strengthen the T cell activity (267, 268). Given that the effectiveness of BsAbs in the therapy of solid tumors appears to be constrained by low tumor infiltration and on-target, off-tumor activity ending in dose limiting toxicity (DLTs), BiTE-armed oncolytic viruses represent an attractive new strategy for enhanced performance (187, 269–272).

### 6.3 Resistance to BsAb therapy

Another concern is the emergence of resistance to BsAb treatment. One typical tactic of resistance is the downregulation or removal of BsAb-specific TAAs on carcinoma cells, which results in cancer immune evasion. ICI resistance is linked to cancer-intrinsic deficiencies in antigen presentation, such as lack of β2 microglobulin needed for MHCI protein production on the cell membrane. Immune checkpoint overexpression, a key component of the immunosuppressive TME, could help with BsAb resistance. Although ICIs have transformed lung carcinoma therapy and are now the standard of care for patients with advanced solid cancers, but the general reaction is still poor, which reveals significant unsatisfied therapeutic needs (37, 273–275). A monovalent IgG1-based BsAb named MEDI5752 was developed to specifically inhibit the PD-1 cascade and CTLA-4 on PD-1^+^ stimulated T cells. Notably, in contrast to the individual PD-1-targeted or CTLA-4-targeted mAbs, MEDI5752 was discovered to promote swift internalization and breakdown of PD-1 and to concentrate selectively in malignancies, generating stronger anti-cancer efficacy (276). OX40/CTLA-4-specific BsAbs are in the preliminary stages of research. Agonist mAbs have been developed to stimulate the immunological costimulatory protein OX40. A number of BsAbs that can selectively attach to OX40 and CTLA-4 to target Tregs in the TME have been invented. These BsAbs are reported to have anticancer impact on bladder, pancreatic, and colon cancers and to work in conjunction with anti-PD-1 antibodies to control colorectal tumor (277, 278).

### 6.4 Side toxicity of BsAbs

The two major issues associated with the cytotoxicity of BsAbs are CRS and neurotoxicity. Exhaustion, headache, and fever are common signs of CRS, as well as more serious symptoms like hypotension, tachycardia, acute respiratory distress syndrome, and circulatory failure. Sequential dosage, premedication with dexamethasone, and SC dosing might reduce the frequency and intensity of CRS (37, 68, 163). The symptoms of neurotoxicity comprise headache, intention tremor, speech difficulties, cognitive problems, and seizures (187). Step dosage and prophylactic corticosteroid usage are among the methods for managing neurotoxicity (37, 279), but there are also ideas to utilize anti-adhesive medicines attempting to avoid neurotoxicities. According to Klinger et al., T-cell adherence to endothelium is a required but inadequate precursor to the emergence of blinatumomab-related neurological negative effects, and inhibiting adherence is a potential mitigating strategy (279).

### 6.5 Large-scale manufacturing of BsAbs

For a number of factors, producing BsAbs is difficult. For instance, challenges with protein production, stability, or the adoption of atypical production techniques may lead to low yields or unsuitable facility design (110). Since the essential methods for regulated Fab-arm interchange are consistent with typical procedures for commercial synthesis of conventional human IgG1, this technology seems to be well suited for large-scale manufacture. The adaptability was validated by the large-scale application of the bench-scale technique, which did not require extensive tuning or result in a decline of output quality (33). CHO cells were used to produce two original antibodies, each of which had a single matching point alteration in the CH3 domain. These antibodies were then produced at a volume of 1000 L utilizing a system fed-batch and purifying procedure created for conventional antibody manufacturing. By combining the two mother molecules in carefully monitored reduction circumstances, the BsAb was produced, featuring an effective Fab-arm interchange of >95% at kilogram scale. By using diafiltration to eliminate the reductant, the disulfide between chains spontaneously reoxidized. In addition to the molecule’s bispecificity, in-depth evaluation showed that the IgG1 structural coherence was preserved, involving functionality and durability (110).

## 7 Conclusion and future perspectives

Recent advances in cancer immunotherapy have highlighted the enormous potential of BsAbs as innovative targeted therapy tools. Over the past two decades, the development of BsAbs has accelerated significantly, benefiting from groundbreaking innovations in antibody formatting. Notably, strategies such as modifying the Fc region have proven effective in mitigating adverse effects (68, 280), while antibody tandem techniques and dimerization enhanced the stability of BsAbs without involving the Fc region (173, 281, 282). However, the paradox between stability and adverse effects associated with the presence or absence of the Fc fragment remains a complex issue requiring further exploration through deliberate design strategies (173). Emerging gene therapies such as genetically engineered cells or nucleic acid drugs hold promise for improving the efficacy and safety paradox.

The landscape of BsAb therapies continues to expand with some preclinical and clinical studies suggesting macrophages and neutrophils as potential therapeutic targets (283). These innate immune cells possess unique cytotoxic properties that can be leveraged to eliminate cancer cells (284). Notably, tumor-associated neutrophils (TANs), especially tumor-associated macrophages (TAMs), are already abundantly present in the TME, precluding the need for external recruitment (285–288). By stimulating these immune effectors *in situ*, BsAb therapies offer the potential for highly efficient tumor cell clearance (289). The strategy of combining BsAbs with other antitumor approaches such as radiotherapy, chemotherapy, mAb, Small-molecule inhibitors or other BsAbs, can overcome the limitations of monotherapy. ATIM-05 (anti-CD3 and anti-EGFR) used prior to radiation/temozolomide, can overcome the highly immunosuppressive glioblastoma (GBM) microenvironment (290). A clinical study (NCT02892123) has shown that ZW25 is well tolerated in combination with chemotherapeutic agents (paclitaxel, capecitabine, or vinorelbine). This approach has excellent and long-lasting antitumor activity in patients with HER2^+^ BC (291). The clinical study (NCT04626635) has demonstrated that RENG7075 used in tandem with cemiplimab boosts the anti-tumour effectiveness of cemiplimab immunotherapy by targeting CD28 with BsAbs (292). Combining BsAb treatment with immune checkpoint blockade provides another avenue for enhanced anti-tumor responses (293). Such combinations take advantage of BsAbs preferential targeting of specific cell types like CD47 and PD-L1 expressing cells to ensure selective cancer cell elimination while protecting normal cells (294, 295). V-aCD3Mu in combination with ICIs can eliminate solid tumors in different cancer models such as B16-F10, 4T1 and CT26 Models (296). The combination of two BsAbs, such as AK117 and AK112 was used in the humanized PD-1 mice models, showing good anti-tumour effects (297). These combined approaches present a promising therapeutic option that provides a wider outlook for BsAbs.

Despite significant progress, BsAb development in oncology remains unfinished. While several BsAbs have gained marketing approval, the rapid emergence of trispecific and multispecific antibodies highlights their dynamic potential (298–301). Robust validation through larger phase II-III clinical trials across cancer types and individualized treatment regimens will be key to fully harnessing the advantages of BsAbs. However, in the realm of solid tumor treatment, BsAbs still face many challenges including but not limited to off-target effects, immune cell penetration, toxicity, resistance mechanisms, manufacturing complexity, and cost (188, 302, 303).

In summary, BsAbs represent a promising direction for designing and developing novel anti-cancer drugs, and their continued R&D will be an integral part of biotherapeutic oncology. Meanwhile, optimizing BsAb pharmacokinetics, manufacturability, deeply understanding factors impacting their penetration and activity in the TME, developing rational combination therapies to improve response rates, and evaluating their engagement with other immune cells like NK cells and CAR-T cells, while elucidating resistance mechanisms, will be critical to advancing BsAbs clinical translation and realizing their anti-tumor potential.

## Author contributions

GS: Conceptualization, Writing – original draft, Writing – review & editing. XG: Writing – original draft, Writing – review & editing. YW: Writing – original draft, Writing – review & editing. YX: Writing – original draft. NX: Writing – original draft, Writing – review & editing.

## Glossary

**Table:**

BsAbs	bispecific antibodies
BsNbs	bispecific nanobodies
TCR-T	T cell receptor T cells
CAR-T	chimeric antigen receptor T cells
ICBs	immune checkpoint blockades
mAbs	monoclonal antibodies
Fab	fragment antigen-binding
EpCAM	epithelial cell adhesion molecule
FcγRs	Fcγ
Fc	fragment crystallisable
IgG	Immunoglobulin G
FcRn	feonatal Fc receptors
Fv	fragment variable
CH	constant heavy chain
CL	constant light chain
DART	dual-affinity retargeting
VH	variable heavy chain
VL	variable light chain
SCFV	single chain variable fragment
VHH	variable heavy-chain segments
VNAR	variable new antigen receptor
hetEHD2	heterodimerizing EH Domain Containing 2
MHD2	IgM heavy chain domain 2
Nbs	nanobodies
EGFR	epidermal growth factor receptor
HER2	human epidermal growth factor receptor 2
Rha	rhamnose
TNF	tumour necrosis factor
IL	Interleukin
PD-L1	programmed cell death-ligand 1
VEGFR1D2	vascular endothelial growth factor receptor type 1 domain 2
KIHs	knobs-into-holes
DutaFab	dual targeting Fab
DVD-Ig	dual variable domain-Ig
FIT-Ig	Fabs-In-Tandem Immunoglobulin
Duobody	controlled Fab-arm exchange technology
TandAb	tandem diabodies
BiTE	bispecific T cell engager
ImmTAC	immune mobilizing monoclonal TCRs against cancer
BriKE	bi-specific killer cell engagers
CD3	cluster of differentiation 3
CEA	carcinoembryonic antigen
Ang2	angiopoietin-2
VEGF	vascular endothelial growth factor
TIM3	T cell immunoglobulin domain and mucin domain-3
FORCE	format chain exchange
SEED	strand-exchange engineered domain
cFAE	controlled Fab-arm exchange
IV	intravenous
IC	intracutaneous
SC	subcutaneous
IM	intramuscular
RMT	receptor-mediated transcytosis
PEG-PLA	polyethylene glycol-lactic-co-glycolic acid
P-gP	P-glycoprotein
PD-1	programmed cell death protein 1
TAA	tumor-associated antigen
HLA	human leucocyte antigen
ICIs	immune checkpoint inhibitors
TCR	T-cell receptor
NHL	non-hodgkin lymphoma
MM	multiple myeloma
AML	acute myeloid leukemia
CHO	Chinese hamster ovary
TDCC	T-cell-dependent cellular cytotoxicity
LAG3	lymphocyteactivation gene 3
DCs	dendritic cells
NK	natural killer
APCs	antigen-presenting cells
LAK	lymphokine-activated killer
CIK	cytokine-induced killer
EAT	*ex vivo* armed T cells
TI	therapeutic indices
ADC	antibody-drug conjugate
NSCLC	non-small-cell carcinoma
EPR	enhanced permeability and retention
MTD	maximum tolerated dose
NRG1	neuregulin 1
ADCC	antibody-dependent cell-mediated cytotoxicity
CDC	complement-dependent cytotoxicity
TME	tumor microenvironment
DLTs	dose limiting toxicity
TAMs	tumor-associated macrophages
